# Lack of chromokinesin Klp-19 creates a more rigid midzone and affects force transmission during anaphase in *C. elegans*

**DOI:** 10.1101/2023.10.26.564275

**Published:** 2023-10-26

**Authors:** Vitaly Zimyanin, Magdalena Magaj, Che-Hang Yu, Theresa Gibney, Basaran Mustafa, Xavier Horton, Karsten Siller, Louis Cueff, Hélène Bouvrais, Jacques Pécréaux, Daniel Needleman, Stefanie Redemann

**Affiliations:** 1 Department of Molecular Physiology and Biological Physics, University of Virginia, School of Medicine, Charlottesville, VA, USA; 2 Center for Membrane and Cell Physiology, University of Virginia School of Medicine, Charlottesville, VA, USA; 3 Department of Cell Biology, University of Virginia School of Medicine, Charlottesville, VA, USA.; 4 Department of Electrical and Computer Engineering, University of California, Santa Barbara, CA, USA; 5 Department of Biology, University of Virginia, Charlottesville, VA, USA; 6 Molecular and Cellular Biology and School of Engineering and Applied Sciences, Harvard University, Cambridge, MA, USA; 7 IT-Research Computing, University of Virginia, Charlottesville, VA, USA; 8 CNRS, Univ Rennes, IGDR (Institut de Génétique et Dévelopement de Rennes) – UMR 6290, F-35000 Rennes, France.; 9 Center for Computational Biology, Flatiron Institute, New York, NY, USA

## Abstract

Recent studies have highlighted the significance of the spindle midzone – the region positioned between chromosomes – in ensuring proper chromosome segregation. By combining advanced 3D electron tomography and cutting-edge light microscopy we have discovered a previously unknown role of the regulation of microtubule dynamics within the spindle midzone of *C. elegans*. Using Fluorescence recovery after photobleaching and a combination of second harmonic generation and two-photon fluorescence microscopy, we found that the length of the antiparallel microtubule overlap zone in the spindle midzone is constant throughout anaphase, and independent of cortical pulling forces as well as the presence of the microtubule bundling protein SPD-1. Further investigations of SPD-1 and the chromokinesin KLP-19 in *C. elegans* suggest that KLP-19 regulates the overlap length and functions independently of SPD-1. Our data shows that KLP-19 plays an active role in regulating the length and turn-over of microtubules within the midzone as well as the size of the antiparallel overlap region throughout mitosis. Depletion of KLP-19 in mitosis leads to an increase in microtubule length in the spindle midzone, which also leads to increased microtubule – microtubule interaction, thus building up a more robust microtubule network. The spindle is globally stiffer and more stable, which has implications for the transmission of forces within the spindle affecting chromosome segregation dynamics. Our data shows that by localizing KLP-19 to the spindle midzone in anaphase microtubule dynamics can be locally controlled allowing the formation of a functional midzone.

## Introduction

Anaphase, the process that separates the duplicated genetic material, consists of two different phases, Anaphase A, a shortening of the chromosome to spindle pole distance, and Anaphase B, an increase in the distance between the spindle poles. The forces that drive chromosome segregation during anaphase A are thought to be generated by depolymerization of kinetochore microtubules, while the forces in anaphase B are thought to rely on a combination of antiparallel sliding of microtubules (MT) between the chromosomes and cortical pulling forces on astral MTs, both driven by motor proteins. In many spindles, both anaphase A and anaphase B occur simultaneously (J Richard McIntosh et al., 2012).

Chromosome segregation in the first mitotic division of the *C. elegans* embryo has long been known to be strongly affected by cortical force generators, a trimeric complex composed of a membrane anchored Gα-protein GOA-1/GPA-16, GPR-1/2 and LIN-5 that recruits dynein, during anaphase. Those force generators generate pulling forces that elongate the spindle (Colombo et al., 2003; Stephan W Grill et al., 2003; S W Grill et al., 2001; Labbé et al., 2004) and which were thought to drive chromosome segregation. As the pole-to-chromosome distance remains constant during this process, Anaphase B was proposed to be the main mechanisms in *C. elegans* (Oegema et al., 2001). However, it was recently shown that spindle elongation and chromosome segregation are two mechanistically distinct processes in *C. elegans* mitosis: spindle elongation results from cortical pulling forces (Colombo et al., 2003; Stephan W Grill et al., 2003; S W Grill et al., 2001; Labbé et al., 2004; Yu et al., 2019), while chromosome segregation is presumably primarily governed by poorly appreciated processes internal to the spindle (Laband et al., 2017; Nahaboo et al., 2015; Yu et al., 2019). Analysis of the midzone in tissue culture cells has confirmed a similar role for the spindle midzone during chromosome segregation in mammalian cells (Yu et al., 2019). However, we still lack significant information about how the spindle midzone generates forces contributing to chromosome segregation during anaphase (Anjur‑Dietrich et al., 2021).

Several conserved proteins localize to the spindle midzone in mammalian cells and *C. elegans,* coordinating its assembly and function. BUB1/BUB-1, a kinetochore protein kinase, was suggested to control the initiation of midzone assembly together with centromere protein CENPF/HCP-1/2 and CLASP/CLS-2. Several publications indicated that the microtubule regulator CLS-2 promotes the *de novo* nucleation of microtubules in the midzone, thus possibly driving the formation of the spindle midzone in *C. elegans* (Edwards et al., 2018; Han & Srayko, 2010; Maton et al., 2015; Nahaboo et al., 2015). In addition, the RAN pathway has been suggested to play a role in the midzone by promoting microtubule assembly or stabilization (Carazo‑Salas et al., 1999; Dasso, 2001; Kalab et al., 1999; Moore, 2001; Ohba et al., 1999; Wilde & Zheng, 1999). The microtubule bundling factors PRC1/ SPD-1 the kinesins KIF4a/ KLP-19 and EG-5/ BMK-1 as well as centralspindlin, a protein complex composed of the kinesin MKLP1/ ZEN-4 and RACGAP1/ CYK-4, then localize to the midzone where they regulate the microtubule organization (Mishima et al., 2002; White & Glotzer, 2012).

A crucial component of midzone stability and function in mammalian cells is PRC1, which plays a central role in crosslinking antiparallel microtubules (Kurasawa et al., 2004; Mollinari et al., 2002; Vernì et al., 2004; Zhu & Jiang, 2005). The absence of PRC1 leads to an inability to establish robust spindle midzones and frequent cytokinesis failures (Kurasawa et al. 2004; Mollinari et al. 2005; Mollinari et al. 2002; Adriaans et al. 2019; do Rosário et al. 2023). In comparison, depletion of SPD-1, the PRC1 homolog in *C. elegans*, equally prevents the formation of a stable midzone, leading to premature spindle rupture. Cytokinesis however is mostly completed, presumably due to contractile ring constriction-driven bundling of astral microtubules at the furrow tip that leads to the formation of a midbody in the absence of a spindle midzone (Hirsch et al., 2022).

PRC1 has been shown to directly interact with various components of the midzone, such as CLASP1, a protein that binds to the plus-ends of microtubules and promotes their growth (Aher et al., 2018; Al‑Bassam & Chang, 2011; Liu & Lampson, 2009; Maton et al., 2015), MKLP1, a subunit of the centralspindlin complex, contributing to the mechanical properties of the midzone (Hutterer et al., 2009; Lee et al., 2015; Mishima & Lee, 2015), and the kinesin KIF4A, which inhibits the elongation of microtubule plus-ends within the midzone (Hu et al., 2011; Nunes Bastos et al., 2013; Subramanian et al., 2013). In *C. elegans* SPD-1 was also shown to directly interact with CYK-4, a component of the centralspindlin complex (Lee et al., 2015), other interactions in *C. elegans* have not yet been assessed.

In vitro experiments on individual microtubules showed that PRC1 and KIF4A accumulate to form dynamic end tags, with the length of these tags being proportional to the concentration of PRC1 and the length of the microtubules (Subramanian et al., 2010, 2013). Mixtures of PRC1, KIF4A, and microtubules also give rise to antiparallel bundles in vitro (Bieling et al., 2010; Hannabuss et al., 2019; Kapitein et al., 2008; Wijeratne & Subramanian, 2018). As microtubules slide apart, the overlap zones between them shorten and eventually reach a steady state, resembling the length of overlap zones within the midzone observed in cells (Bieling et al., 2010; Hannabuss et al., 2019). PRC1 has been shown to generate frictional forces that counteract microtubule sliding, aligning with the braking function of midzones observed in cells (Gaska et al., 2020; Pamula et al., 2019; Saunders et al., 2007).

In *C. elegans*, depletion of either SPD-1/PRC1, BMK-1/ EG5 or ZEN-4/ MKLP1 results in rapid chromosome segregation and often breakage of the spindle midzone during anaphase. The spindle rupture can be suppressed by depletion of cortical pulling forces, suggesting that those midzone proteins are required for the mechanical integrity of the midzone during its elongation (Lee et al., 2015; Maton et al., 2015; Nahaboo et al., 2015). These observations strongly indicate a dual role for the midzone in anaphase. Firstly, it acts as a stabilizing element, countering intense cortical pulling forces and regulating the rate of chromosome segregation. Secondly, it functions as a force generator, facilitating chromosome segregation. This complex interplay underscores the challenge of balancing the conflicting demands on the midzone during cell division. The mechanisms how the spindle midzone achieves this have remained elusive.

Combining light and electron microscopy we show that the chromokinesin KLP-19 regulates the dynamics, stability and length of microtubules in the spindle midzone during anaphase. In absence of KLP-19 microtubules are longer and more stable and thus can form more interactions with neighboring microtubules. This leads to the formation of a stiffer microtubule network, which makes the spindle more resistant to cortical pulling forces but at the same time interferes with the fidelity of chromosome segregation.

## Results

### Microtubule overlap in the midzone remains constant throughout anaphase

**Figure 1.**
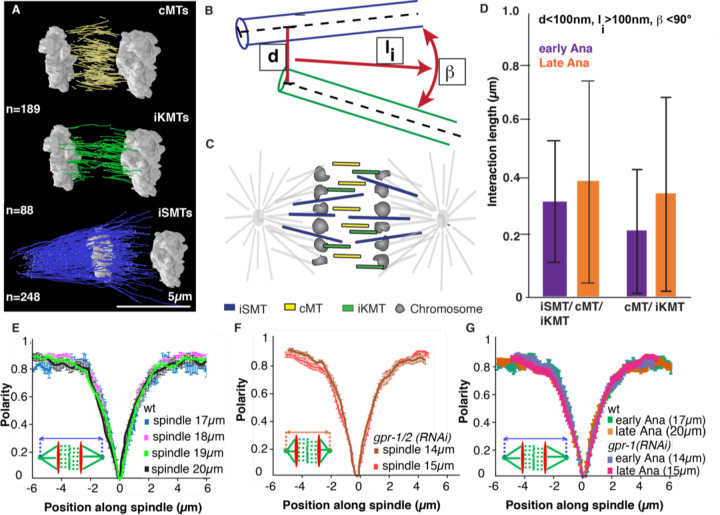
The midzone is composed of different subclasses of microtubules. **A.** Reconstructions of microtubules in the spindle midzone in anaphase of the C. elegans one-cell embryo obtained by 3D electron tomography. The microtubules are grouped into three different groups based on the position of their ends. cMTs (yellow, central MTs) both ends between chromosomes, iKMTs (green, inter kinetochore MTs), both ends between chromosomes, one end touching chromosome surface and iSMTs (blue, interdigitating spindle MTs), only one end between the chromosomes. Number of microtubules is indicated, scale bar 5µm. **B.** Cartoon showing the different microtubule subclasses as determined by electron tomography inside a spindle. **C.** Cartoon of a microtubule-microtubule interaction showing the different parameters used to define interactions. d = Center to center distance between two microtubules, l_i_ = length of interaction, β= orientation angle between the microtubules. **D.** Plot of the interaction length l_i_ between microtubules of the different microtubule subclasses for microtubules with d= 100nm, minimum l_i_ = 100nm and b = 90° for 3 different datasets throughout anaphase, chromosome distance as an indicator for progression through anaphase is provided. Error bars are sem. **E.** Polarity plot of microtubules in the spindle throughout anaphase obtained by the combination of SHG and TP microscopy. 0 on the x-axis represents the spindle center. Spindle length is indicated as a measure of progression throughout anaphase. **F.** Polarity plot of microtubules in the spindle throughout anaphase after *gpr-1/2 (RNAi)* obtained by SHG and TP microscopy combined. **G.** Overlay of polarity plot of microtubules in the spindle throughout anaphase in wild type embryos and embryos after *gpr-1/2 (RNAi)*.

An active role of the spindle midzone in promoting and restricting chromosome segregation requires a very tight regulation of its structure and dynamics. Outward sliding of antiparallel midzone microtubules is thought to be the main regulator of midzone driven chromosome segregation. We used 3D electron tomography to reconstruct the spindle midzone in anaphase (Fig. 1A). As previously reported (Yu et al., 2019) these reconstructions show that some microtubules in the midzone seem to originate from the region between chromosomes and poles (iSMTs= inter spindle microtubules), while other microtubules (central microtubules= cMTs, iKMTs= inner kinetochore microtubules) have both ends between the segregating chromosomes, occasionally contacting the inner-surface of the chromosomes, and potentially nucleating between the chromosomes. Interestingly, only 1 or 2 microtubules extended all the way from pole to pole.

To quantify changes in the interaction of microtubules in the spindle midzone throughout anaphase in the one-cell stage *C. elegans* embryo we quantified the length of interactions between individual microtubules in 3 tomographic reconstructions of mitotic spindles at different stages in anaphase defined by the distance between the segregating chromosomes. The interactions were defined by a maximum center-to-center distance of 100 nm between interacting microtubules, based on our previous data on the spindle network (Redemann et al., 2017) as well as the reported sizes of microtubule-associated proteins or molecular motors (Mastronarde et al., 1993; McDonald et al., 1992; Peterman & Scholey, 2009). We further required a minimum interaction length of 100 nm (based on the detection limit of microtubules, which is 100nm) and an orientation angle of 0–90° between the two microtubules (Fig. 1B, C). Our analysis of microtubule overlap in the midzone (Fig. 1D) showed that the average length of the interactions between midzone microtubules is 370 nm ± 30 nm in early anaphase (chromosome distance 1.5µm), 380 nm ± 20 nm in mid anaphase (distance 4µm) and 420 nm ± 10 nm in late anaphase (distance 5.5µm). This data shows that the interaction length of microtubules increased by approximately 50 nm between MTs from early to late anaphase, while the size of the central spindle, defined by the distance between the segregating chromosomes, increased by ~4μm (80x more) at the same time (Fig. 1D). This data suggests that the microtubule overlap in the C. elegans midzone does not undergo major changes throughout anaphase, in contrast to previously reported data in mammalian cells (Eggert et al., 2006; Euteneuer & McIntosh, 1980; Heidemann et al., 1980; Mastronarde et al., 1993; J R McIntosh et al., 1975; Saxton & McIntosh, 1987; Snyder & McIntosh, 1975).

As our EM data is based on a relatively low sample number and because it is difficult to determine the microtubule polarity in our tomographic reconstructions, we used second harmonic generation (SHG) microscopy and two photon florescence (TP) microscopy simultaneously to measure the polarity of the collective microtubules throughout the spindle in vivo (Yu et al., 2014). SHG is a nonlinear optical process in which highly polarizable, non-centrosymmetric materials emit photons with half the wavelength of incident light. When an array of microtubules is imaged with SHG microscopy, the resulting SH signals depend on both the polarity of the microtubule array and the density of microtubules within a focal volume. The readout of microtubule density can be obtained using TP microscopy and was used to compute the polarity of microtubules, information carried by the SHG signals (Yu et al., 2014). The polarity of microtubules ranges from 0 to 1 continuously: 1 corresponds to parallel microtubules with all the plus ends pointing in the same direction, and 0 corresponds to antiparallel microtubules with equal number of plus ends pointing in the opposite direction. Measurements by SHG and TP in wild type embryos showed that microtubules have a constant antiparallel overlap length in the central spindle (Fig. 1E), indicated by the constant width of the polarity curve during anaphase, consistent with the results from our tomography (Fig. 1D). To test the potential impact of cortical pulling forces on the size of the antiparallel microtubule region in the midzone we repeated the experiment in embryos depleted of GPR-1/2. The size of the antiparallel region did not change in the absence of pulling forces (Fig. 1F), showing that the antiparallel overlap region is constant throughout anaphase and independent of pulling forces (Fig. 1G). These results suggest that in addition to microtubule sliding, some microtubule polymerization or re-arrangement must appear during chromosome segregation in the midzone to maintain the overlap. It is noteworthy that while the overlap length remained constant in absence of pulling forces, as the spindles are shorter after *gpr-1/2 (RNAi)*, the percentage of microtubule overlap in those spindles increased.

### Removal of KLP-19 protects midzone from pulling forces and rescues *spd-1* (RNAi) induced spindle rupture.

The interaction of the kinesin KIF4a with the microtubule bundling protein PRC1 has been suggested to be key to the regulation of microtubule overlap. Based on *in vitro* data it has been proposed that PRC1 recruits KIF4a to the microtubules in the spindle midzone, where it regulates microtubule dynamics and midzone length, possibly through its ability to inhibit microtubule plus-end growth (Kurasawa et al., 2004; Zhu & Jiang, 2005).

**Figure 2.**
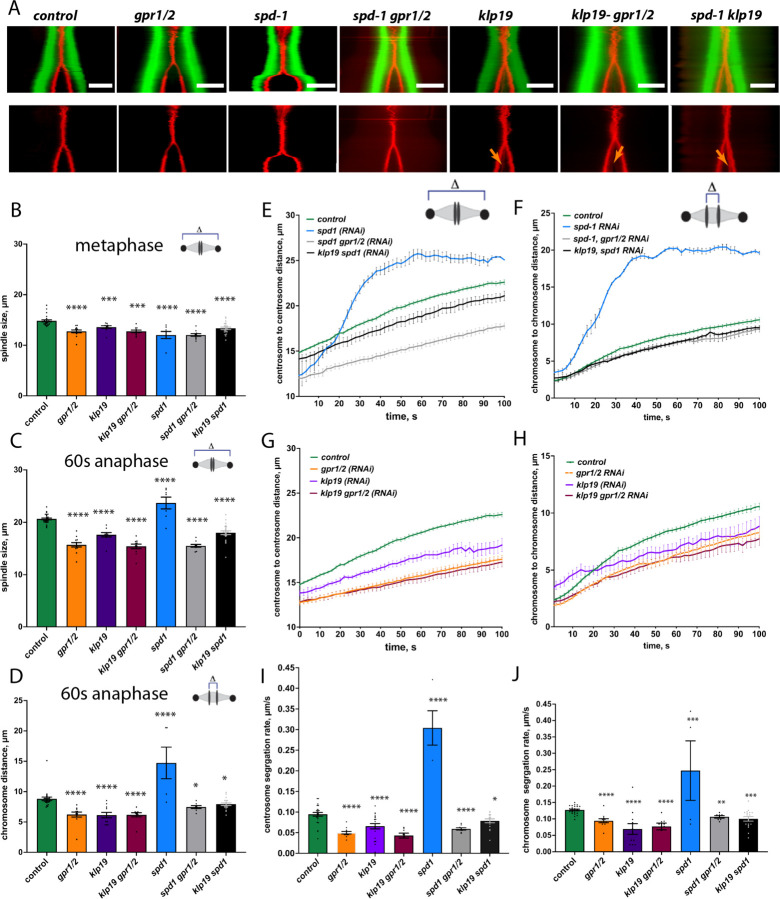
Depletion of KLP-19 and SPD-1 affect spindle dynamics. **A.** Kymographs of *C. elegans* one-cell embryos from metaphase to anaphase in control and after *gpr-1/2, spd-1, spd-1/gpr-1/2, klp-19, klp-19/gpr-1/2 and spd-1/klp-19 RNAi* obtained by light microscopy. Microtubules are labeled with GFP, histone with mCherry. Orange errors point to lagging chromosomes. Scale Bar 10µm. **B.** Bar plot of the spindle length at metaphase in control embryos and after different RNAi treatments. **C**. Bar plot of the spindle length 60s after anaphase onset in control embryos and after different RNAi treatments. **D**. Bar plot of the Chromosome distance 60s after anaphase onset in control embryos and after different RNAi treatments. **E.** Plot of pole-to-pole distance throughout anaphase in control embryos (green), after *spd-1 (RNAi)* (blue), *klp-19/ spd-1 (RNAi)* (black) and *spd-1/ gpr-1/2 (RNAi)* (grey). **F.** Plot of chromosome distance throughout anaphase in control embryos (green), after *spd-1 (RNAi)* (blue), *klp-19/ spd-1 (RNAi)* (black) and *spd-1/ gpr-1/2 (RNAi)* (grey). **G.** Plot of pole-to-pole distance throughout anaphase in control embryos (green), after *klp-19 (RNAi)* (purple), *gpr-1/2 (RNAi)* (orange) and *klp-19/ gpr-1/2 (RNAi)* (maroon). **H.** Plot of chromosome distance throughout anaphase in control embryos (green), after *klp-19 (RNAi)* (purple), *gpr-1/2 (RNAi)* (orange) and *klp-19/ gpr-1/2 (RNAi)* (maroon). **I.** Bar Plot of pole-to-pole segregation rate throughout anaphase in control embryos and embryos after different RNAi treatments **J.** Bar Plot of chromosome segregation rate throughout anaphase in control embryos and embryos after different RNAi treatments. Error Bars are sem, significant differences (t-test) are indicated by asterisk.

During *C. elegans* mitosis the PRC1 homolog, SPD-1, localizes to the spindle midzone in anaphase (Verbrugghe & White, 2004). Our analysis of spindle elongation (pole to pole distance) and chromosome segregation (chromosome distance) showed that metaphase spindles were shorter after *spd-1 (RNAi)* as well as SPD-1/ GPR-1/ 2 co-depletion (Fig. 2A–C, Suppl. Fig. 1A). Depletion of SPD-1 alone lead to increased rates of spindle elongation and chromosome segregation due to spindle rupture during anaphase (Fig. 2 E, I, Suppl. Fig. 1), as previously reported (Verbrugghe & White, 2004). Co-depletion of GPR-1/ 2 prevented spindle rupture and the rate of spindle elongation was significantly decreased in comparison to control embryos as well as *spd-1 (RNAi)* alone (Fig. 2E, I, Suppl. Fig. 1). The rate of chromosome segregation in *spd1/ gpr-1/ 2 (RNAi)* embryos was also reduced (Fig 2J).

Depletion of the KIF4a *C. elegans* homolog KLP-19 resulted in severe chromosome segregation defects and showed a strong disruption in metaphase plate and kinetochore alignment and its overall organization, including reduced compaction of chromosomes, as well as presence of multiple lagging chromosomes during segregation (Suppl. Fig 2A) (Powers et al., 2004). We could confirm a role for KLP-19 during mitosis, in generating polar-ejection forces that move chromosomes away from the spindle pole towards the metaphase plate during congression (Suppl Fig 3) (Powers et al., 2004). The observed chromosome missegregation had been associated with errors in the correct formation of end-on connections of MTs to kinetochores (Powers et al., 2004).

Detailed analysis of the chromosome segregation and spindle elongation rates showed that *klp-19 (RNAi)* leads to the formation of shorter metaphase spindles (Fig 2B–D, Suppl. Fig. 1), and a reduction in the pole-to-pole and chromosome segregation rates (Fig 2G, H). To further analyze the role of KLP-19 and SPD-1 we co-depleted both proteins by RNAi. Chromosome congression errors and subsequent chromosome missegregation still occurred upon co-depletion (Suppl Fig 1A, Suppl. Fig 2A). However, to our surprise co-depletion of SPD-1 and KLP-19 did not lead to spindle rupture as observed by *spd-1 (RNAi)* alone (Fig. 2E, Suppl. Fig. 1, Suppl. Fig. 2). In contrast, spindle rupture was prevented and spindle elongation rates, as well as chromosome segregation rates were slightly reduced in comparison to control embryos (Fig 2A–D). To exclude potential effects due to decreased RNAi efficiency in double RNAi experiments we also depleted KLP-19 in the SPD-1 temperature sensitive mutant (oj5). This also showed a prevention of spindle breakage after *klp-19 (RNAi)* (Fig Suppl 4).

To determine if the observed effect was specific to SPD-1 and KLP-19 co-depletion, we co-depleted KLP-19 together with other proteins that had previously been shown to lead to spindle rupture (S W Grill et al., 2001; Mishima et al., 2002, 2004; Raich et al., 1998; Srayko et al., 2005). To this end we tested ZEN-4/ MKLP1 as well as the MT depolymerase KLP-7, the *C. elegans* homolog of MCAK. In all cases co-depletions with KLP-19 prevented the spindle breakage (Suppl. Fig.1B, Suppl. Fig. 5). As spindle rupture is thought to be caused by the cortical pulling forces, these results suggest that depletion of KLP-19 could affect the transmission of these forces to the spindle, thus preventing its breakage. As potential alternatives KLP-19 could directly affect pulling forces, could affect the organization of astral MTs and their interaction with cortical force generators, or global change the architecture of the spindle network and midzone.

### Cortical pulling forces are still active in *klp19 (RNAi*) conditions.

Rescue of the midzone formation in SPD-1 KLP-19 double RNAi conditions could occur due to downregulation of cortical forces comparable to downregulation of GPR-1/2.

**Figure 3.**
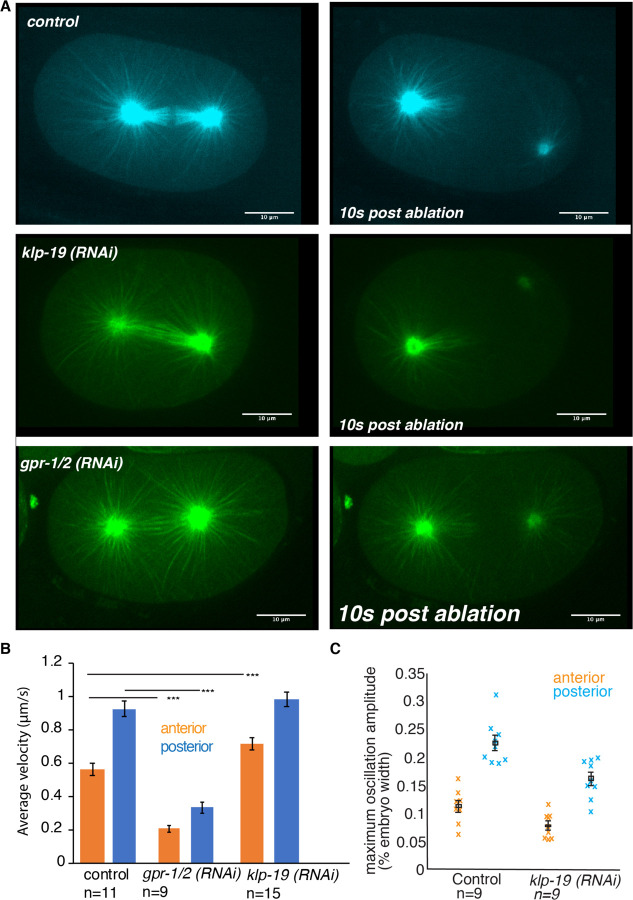
KLP-19 depletion does not directly affect cortical pulling forces. **A.** Stills of embryos before (left) and 10 s after (right) laser microsurgery severing the posterior centrosome from the spindle in control (top), *klp-19 (RNAi)* and *gpr-1/2 (RNAi)* treated embryos. **B.** Plot of the velocity of the anterior (orange) and posterior (blue) centrosome after laser microsurgery in control (top), *klp-19 (RNAi)* and *gpr-1/2 (RNAi)* treated embryos. **C.** Maximum Oscillation amplitude of the anterior (orange) and posterior (blue) centrosome during anaphase in control and klp-19 (RNAi) embryos. Error bars are sem, Scale bar is 10µm.

To determine potential direct effects on pulling forces in response to *klp-19 (RNAi)* we conducted laser-ablation experiments, in which we severed the posterior centrosome by cutting the microtubules between centrosome and chromosomes (Fig 3A). Subsequent quantification of the velocity of the posterior centrosome showed increased velocities in *klp-19 (RNAi)* in comparison to control, while *gpr-1/ 2 (RNAi)* led to a significant reduction in centrosome velocity (Fig 3B). Consistently, while anaphase oscillations of the posterior centrosome were mildly reduced in *klp-19(RNAi)* (5.2 ± 1.1 µm (mean ± SD, *N* = 9)) compared to control conditions (7.1 ± 1.2 µm (*N* = 9, *p* = 00.4)) (Fig. 3C), the centering forces were increased as measured through the stability of the metaphase spindle; the filtered standard deviation of the spindle-center position along the transverse axis, measured between 165 s and 40.7 s before anaphase onset, was SD_y_=32.0 ± 2.5 nm (mean in quadrature ± SD, *N* = 7) upon *klp-19(RNAi)* compared to 40.1 ± 1.9 nm (*N* = 9) in control. Overall, centering is only moderately affected in *klp-19(RNAi).* This suggests that net cortical pulling forces are only modestly affected *by klp-19(RNAi)* and are thus still present in the SPD-1 and KLP-19 co-depletion. Based on this we hypothesized that the transmission of cortical pulling forces to the spindle is altered upon *klp-19 (RNAi)*. In order to further test this, we depleted KLP-19 in embryos that express a codon adapted version of GPR-1/2 leading to increased expression levels and thus display excessive cortical pulling forces resulting in spindle rupture in 33% of the one-cell embryos, often prior to the formation of a metaphase spindle (Redemann et al., 2011). Depletion of KLP-19 completely prevented spindle rupture in those embryos and led to a decrease in pole and chromosome separation rates, thus suggesting that loss of KLP-19 protects spindles from cortical pulling forces (Suppl. Fig. 6).

As spindles depleted of KLP-19 seemed to be more resistant against cortical pulling forces, we hypothesized that *klp-19 (RNAi)* could affect the organization and dynamics of microtubules in the midzone, making it more stable and robust.

### KLP-19 regulates the microtubule overlap independently of the microtubule bundling protein SPD-1

**Figure 4.**
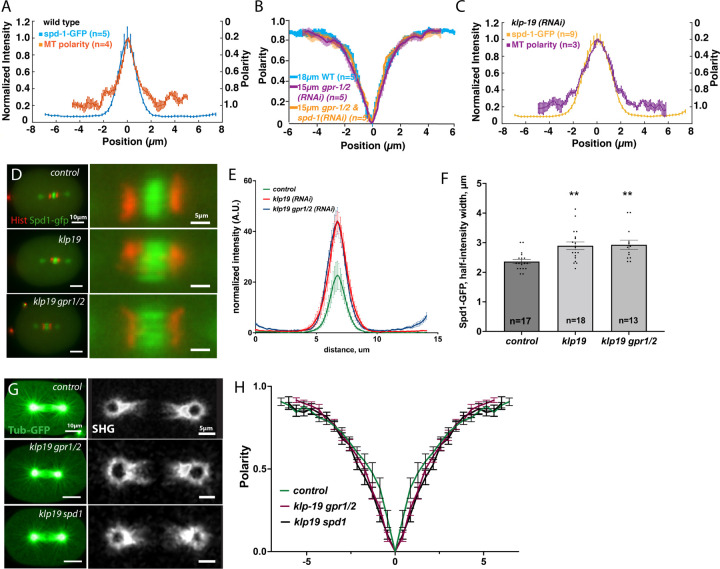
KLP-19 regulates the microtubule overlap length in the spindle midzone. **A.** Plot showing the normalized intensity (axis on the left) of SPD-1 GFP (blue) along the spindle axis (0 is the spindle center on x axis) in anaphase and the microtubule polarity (right axis, orange) along the spindle axis (right y-axis) in control embryos. “0” is the spindle center **B.** Plot showing the polarity of microtubules along the spindle axis in control embryos (blue), embryos depleted of pulling forces by *gpr-1/2 (RNAi)* (purple) and embryos after *spd-1/ gpr-1/2 (RNAi)* (orange). “0” is the spindle center **C.** Plot showing the normalized intensity (left y-axis) of SPD-1 GFP (yellow) along the spindle axis (0= spindle center) in anaphase and the polarity of microtubules (right y-axis, purple) along the spindle axis in embryos after *klp-19 (RNAi)*. **D. Left:** Stills of anaphase spindles labeled with γ-tub::mCherry, histone::mCherry and SPD-1::GFP in control embryos (top) and embryos after *klp-19 (RNAi)*. Right: Zoom into the spindle midzone. Scale bar 5µm. **E.** Plot of the normalized SPD-1::GFP intensity along the midzone 60s after anaphase onset in control embryos and after *klp-19 (RNAi)*. **F.** Bar plot of the SPD-1 GFP half-intensity width in control, *klp-19 (RNAi)* and *klp-19/gpr-1/2 (RNAi)* **G.** Left: Two-photon microscopy images of β-tubulin::GFP in control (top), *klp-19/ gpr-1/2 (RNAi)* and klp-19/ spd-1 (RNAi) treated embryos. Right: Corresponding SHG images. Scale Bars are 10µm left, 5µm rigt images **H.** Plot of the polarity of microtubules along the spindle axis (0= spindle center) of control embryos and after *klp-19/ gpr-1/2 (RNAi)* and *klp-19/ spd-1 (RNAi).* Error bars are sem.

PRC-1 has been suggested to recruit KIF4a to the spindle midzone in mammalian cells and in vitro, where both proteins interact to regulate the microtubule overlap (Bieling et al., 2010; Hu et al., 2011; Subramanian et al., 2010, 2013). To assess the effect of *spd-1 (RNAi)* and *klp-19 (RNAi)* on the microtubule overlap in the midzone we used SHG and TP microscopy. Previous studies showed that SPD-1 localizes to and is required for the integrity of antiparallel MTs in the midzone (Verbrugghe & White, 2004). To further explore this, we studied the localization of SPD-1 in the one-cell *C. elegans* embryos. By correlating the localization of SPD-1-GFP with microtubule polarity, using a combination of 2-Photon and SHG microscopy, we found that SPD-1 indeed localizes to the region of antiparallel microtubules in *C. elegans* indicated by the overlapping profiles (Fig 4A, note: the y-axis of the microtubule polarity has been inverted for easier viewing*)*. This localization could suggest a potential role for SPD-1 in regulating overlap. To test this, we analyzed microtubule polarity in absence of SPD-1. As depletion of SPD-1 prevents the formation of a midzone in wild type embryos due to the extensive cortical pulling forces, we measured the microtubule polarity in spindles of *C. elegans* embryos co-depleted of SPD-1 and GPR-1/2. We could not detect any changes in the microtubule overlap in the central spindle, indicated by the overlapping polarity profiles, suggesting that SPD-1 is not required in determining the profile of microtubule polarity when the pulling force is greatly reduced (Fig. 4B).

Since the ratio of PRC1/KIF4A controls the extent of antiparallel microtubule overlap (Hannabuss et al., 2019; Kurasawa et al., 2004; Wijeratne & Subramanian, 2018), we measured the localization of SPD-1 as well as the microtubule polarity in embryos depleted of KLP-19. This showed an approximately 2-fold increase in SPD-1-GFP distribution in KLP-19-depleted embryos (Fig. 4C–E), consistent with previous results (Kurasawa et al., 2004). In agreement with this we found that the antiparallel microtubule region increases after KLP-19 depletion, indicated by an increase in width of the polarity profile measured by SHG and TP signals (Fig. 4C–F). Next, we co-depleted KLP-19 and SPD-1 and quantified the profile of microtubule polarity. We found that the width of the antiparallel microtubule overlap region is increased in these embryos in comparison to wildtype and comparable to the KLP-19 depletion or its co-depletion with GPR-1/2 (Fig 4D–F). In addition, we quantified the SPD-1 region in embryos co-depleted of ZEN-4 and KLP-19. In these embryos the width of the SPD-1 signal was also increased (Suppl Fig. 7) This data shows that KLP-19 regulates the size of the antiparallel microtubule overlap and thus narrows the distribution of SPD-1, confirming a role for the *C. elegans* KLP-19 in the spindle midzone in addition to its previously reported role during congression (Powers et al., 2004).

**Figure 5:**
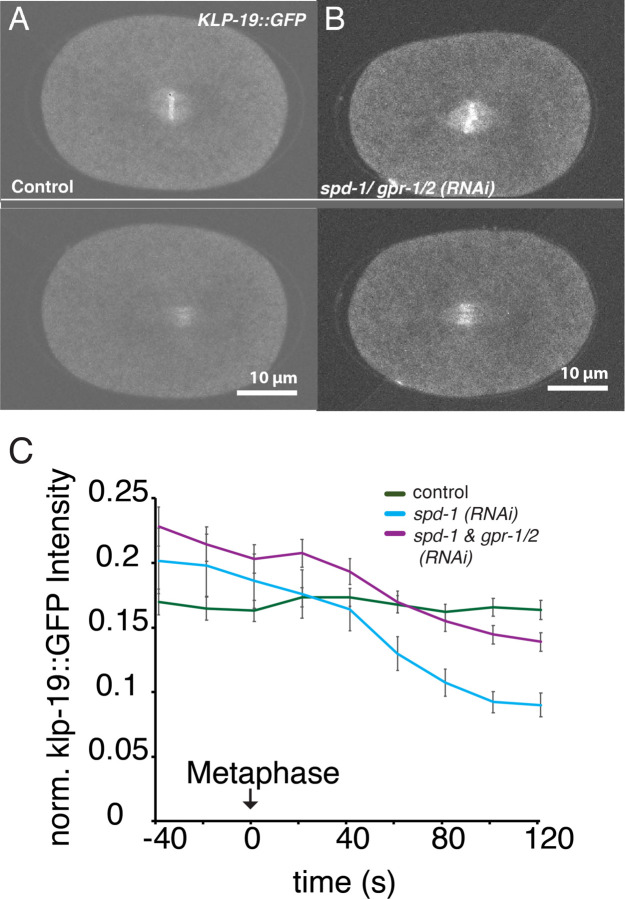
Midzone localization of KLP-19 is independent of SPD-1. **A.** Stills of control embryos in metaphase (top) and anaphase (bottom) expressing endogenously tagged KLP-19 GFP.**B.** Stills of embryos in metaphase (top) and anaphase (bottom) expressing endogenously tagged KLP-19 GFP after *spd-1/gpr-1/2 (RNAi)*. **C.** Plot of KLP-19 GFP intensity in the spindle midzone over time in control embryos, embryos treated with *spd-1 (RNAi)* and *spd-1/ gpr-1/2 (RNAi)*. Error Bars are sem, Scale Bar 10µm.

Based on previous publications we assumed that depletion of SPD-1 should reduce or prevent the localization of KLP-19 to the midzone in *C. elegans*. However, this is inconsistent with the observed effect on microtubule overlap and the different phenotypes upon double depletion. To follow up on this observation we generated a worm strain expressing GFP tagged KLP-19 using CRISPR technology. This strain allowed us to monitor the localization of KLP-19 throughout mitosis. We found that KLP-19 is initially enriched in the pro-nuclei and localizes to chromosomes (Fig. 5A). Upon Nuclear envelope breakdown (NEBD) KLP-19 localizes to chromosomes as well as spindle microtubules but could not be detected on centrosomes or astral microtubules. At anaphase onset KLP-19 transitions from the chromosomes to the spindle midzone (Fig 5A). To determine the dependency of KLP-19 midzone localization on SPD-1 we monitored the localization of KLP-19 GFP during mitosis in *spd-1 (RNAi)* and *spd-1/ gpr-1/ 2 (RNAi)* embryos. While the KLP-19 GFP signal in the spindle midzone was mainly lost after *spd-1 (RNAi),* due to spindle rupture that prevented the formation of a midzone (Fig. 5B), the localization of KLP-19 to the spindle midzone in *spd-1/ gpr-1/ 2 (RNAi)* was unchanged, suggesting that KLP-19 localizes to the spindle midzone independently of SPD-1 in *C. elegans* (Fig. 5B). Likewise, localization to chromosomes was unchanged in either RNAi treatment (Fig 5B).

To further establish how the localization of KLP-19 to the spindle midzone is regulated we depleted the kinetochore proteins BUB-1, HCP-3, HCP-4, NDC-80 and AIR-2. We found that depletion of AIR-2, NDC-80 and HCP-3 increased the amount of KLP-19 on chromosomes in metaphase (Suppl Fig. 8), while depletion of BUB-1 led to a reduction of KLP-19. In anaphase, depletion of AIR-2 and BUB-1 significantly reduced KLP-19 in the midzone. We also detected an increase in KLP-19 in the midzone after depletion of NDC-80, however as chromosome segregation fails in these embryos it is very likely that the increase reflects the amount of KLP-19 on the chromosomes and not in the midzone.

### KLP-19 regulates microtubule dynamics in the spindle midzone.

**Figure 6.**
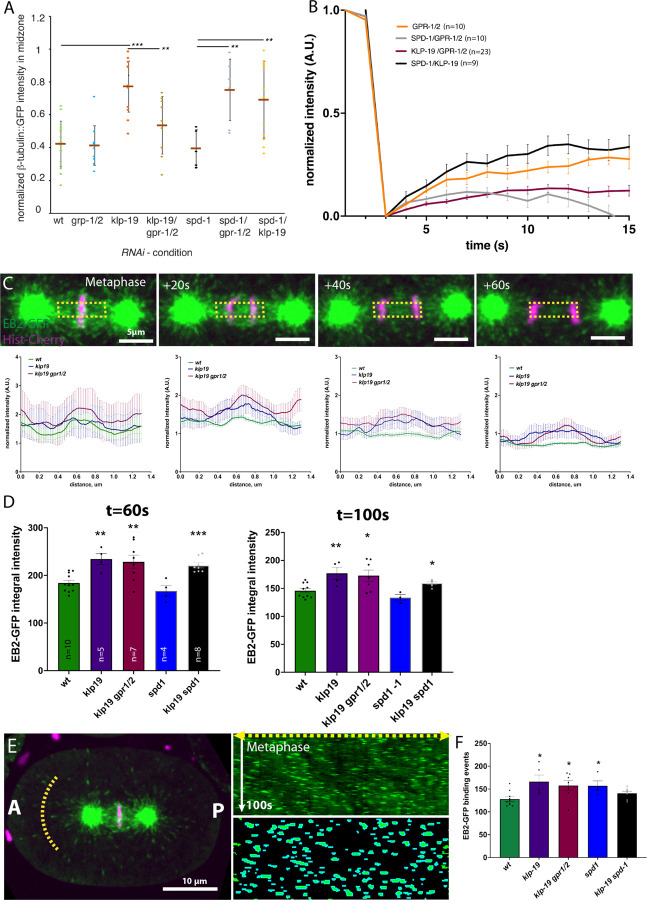
KLP-19 affects microtubule dynamics. **A.** Plot of the normalized β-tubulin::GFP intensity in the spindle midzone 60s after anaphase onset in control embryos and after *gpr-1/2 (RNAi)*, *klp-19 (RNAi)*, *klp-19/gpr-1/2, spd-1 (RNAi)*, *spd-1/gpr-1/2 (RNAi)*, and *spd-1/klp-19 (RNAi).* **B**. Plot of Microtubule fluorescence recovery over time after photobleaching (FRAP) in the spindle midzone in anaphase in control embryos and embryos after *gpr-1/2 (RNAi)*, *spd-1/gpr-1/2 (RNAi)*, *klp-19/gpr-1/2 (RNAi)*and *spd-1/klp-19 (RNAi)*. **C.** Stills of a C. elegans embryo in anaphase expressing EBP-2::GFP and Histone::mCherry. Yellow box indicates the area used for intensity profile measurements shown in the corresponding plots below. **D.** Bar plot showing the normalized intensity of EBP-2::GFP signal in the spindle midzone in control embryos and after *klp-19 (RNAi), klp-19/ gpr-1/2 (RNAi), spd-1 (RNAi)* and *spd-1/ klp-19 (RNAi)* 60s after anaphase onset (left) and 100s after anaphase onset (right). **E.** Left: Still of an embryo expressing EBP-2::GFP and mCherry Histone. Anterior and posterior pole are indicated. Yellow half-circle on the posterior indicates the region of EBP-2 signal quantification. Middle, top: Kymograph of the Yellow line, bottom: Segmented EBP-2 comets. Right: Bar plot showing the quantification of EBP-2 comets at the yellow line over 100s. 4.5µm length segment of the line was analyses for binding events.

While pulling forces act on centrosomes, the forces are also acting on the midzone as a stable midzone is required to resist pulling forces. Since KLP-19 localizes to the spindle midzone and depletion of KLP-19 affects the antiparallel microtubule overlap, we reasoned that the transmission of the pulling forces might be altered in the midzone. Analysis of microtubule intensity in the spindle midzone after *klp-19 (RNAi)* showed an increase in intensity, suggesting a potential increase in microtubule number or density (Fig 6A). Based on the proposed function of KIF4A in stalling microtubule plus-end growth, we speculated that depletion of KLP-19 could affect microtubule dynamics in *C. elegans.* Our previous data on microtubule dynamics in wild type midzones showed that microtubules in the center of the midzone turn-over within 20s, while microtubules near the chromosomes did not turn-over during the course of anaphase (Yu et al., 2019). Analysis of microtubule turn-over after *klp-19 (RNAi)* showed that microtubule turn-over near the chromosomes was even slower in comparison to control (Fig. 6B).

To determine effects on microtubule growth we imaged embryos expressing GFP-tagged EBP-2, which localizes to growing microtubule plus-ends. Quantification of the EBP-2 signal showed an increase in EBP-2 intensity in the midzone upon *klp-19 (RNAi)*, suggesting increased microtubule polymerization (Fig. 6C, D). This result is consistent with the proposed stalling of microtubule growth by KLP-19 (Girão et al., 2020; Hu et al., 2011). The effect on microtubule dynamics could be restricted to the midzone or affect global microtubule dynamics. Quantification of EBP-2 binding events on astral microtubules in anaphase showed an increase in embryos depleted of KLP-19 (Fig. 6E). Interestingly, we also detected an increase after *spd-1 (RNAi)*. As our data showed an increase in tubulin intensity, a decrease in microtubule turn-over and an increase in EBP-2 binding events after *klp-19 (RNAi)* we conclude that KLP-19 globally regulates microtubule dynamics during mitosis in *C. elegans* possibly by reducing polymerization and eventually promoting turn-over by inducing depolymerization. As the presence of KLP-19 could destabilize the astral microtubule tips, and thus reduce the transmission of pulling forces, we compared its effect with depleting the depolymerase KLP-7. We first measured the stability of the metaphase spindle as a read out for centering forces in *klp-19 (RNAi)* and *klp-7 (RNAi)* and found that the centering forces are only moderately effect in KLP-19 depleted embryos (SD_y_=32.0 ± 2.5 nm (N=7) in contrast to KLP-7 depleted embryos (SD_y_=16.9 ± 0.6 nm (*N* = 9)). In addition, *klp-7 (RNAi)* leads to spindle rupture, due to increased microtubule interactions with force generators, which is in stark contrast to the effect of *klp-19 (RNAi)*. In summary, this suggests that the effect on astral microtubule dynamics does not affect the transmission of pulling forces.

**Figure 7.**
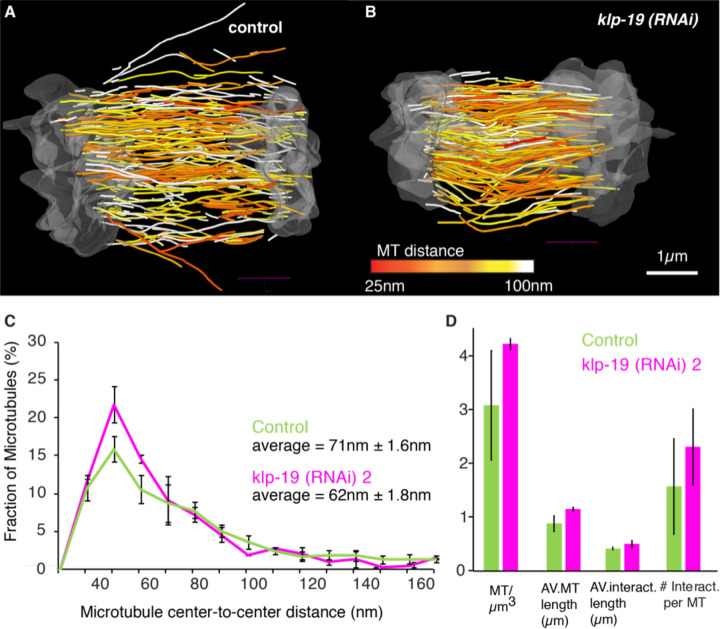
KLP-19 depletion leads to increased microtubule length and interactions. **A.** 3D tomographic reconstruction obtained by electron tomography of control (left) and *klp-19 (RNAi)* embryos showing only microtubule in the spindle midzone. Microtubules are color coded according to the nearest distance to a neighboring microtubule, with red being 25nm and white larger than 100nm. Scale bar is 1µm **B.** Plot of the fraction of microtubule interactions showing a certain microtubule center-to-center distance. Average microtubule distance is provided in the plot. **C.** Bar plot of different average parameters for microtubules in control (green) and *klp-19 (RNAi)-*treated embryos.

To quantify the effect of KLP-19 depletion on microtubule length we used 3D tomography to analyze the microtubule arrangement in the spindle midzone in response to *klp-19 (RNAi)*. (Fig 7, Suppl. Fig. 9). Quantification of microtubule number, length and the interaction between microtubule in the midzone in the tomographic reconstructions revealed an increase in the average microtubule number from 3.1 microtubule/µm^3^ in control to 4.3 microtubule/µm^3^ and an increase in average microtubule length from 0.88 µm ± 0.14 µm in control to 1.15 µm ± 0.03 µm after *klp-19 (RNAi)*. In addition, we also quantified the average distance between microtubules in the reconstructions, which was 71nm ± 2nm in control and 62nm ± 2nm after KLP-19 depletion. The microtubule overlap length changed from 0.42 µm ± 0.02 µm in control to 0.50 µm ± 0.07 µm after *klp-19 (RNAi)*.

In summary this data suggests that KLP-19 is involved in regulating the microtubule length and interaction and thus the overlap in the central spindle by affecting microtubule dynamics. This finding also suggests that the regulation of microtubule overlap and dynamics affects the force transmission within the spindle.

### Robustness of the midzone affects half-spindle length and spindle-length fluctuations.

Most of the chromosome segregation in *C. elegans* was suggested to be driven by anaphase B, a separation of spindle poles, governed by cortical pulling forces on astral microtubules. The distance between spindle poles and chromosomes remains mostly constant during anaphase, suggesting that Anaphase A is not very prominent in the one-cell *C. elegans* embryo. (Oegema *et al.* 2001).

**Figure 8.**
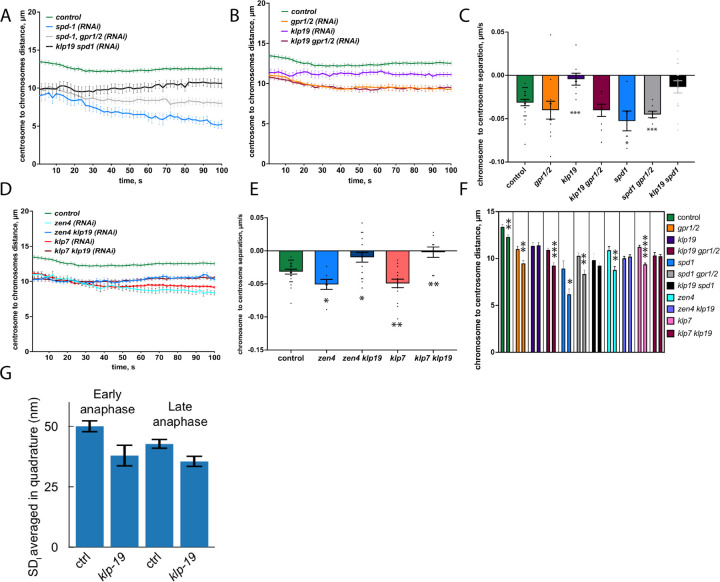
KLP-19 depletion affects half-spindle length and spindle fluctuations. **A.** Plot of the centrosome to chromosome distance (half-spindle length, both halves combined) throughout anaphase for control embryos and embryos treated with *spd-1 (RNAi), spd-1/ gpr/1/2 (RNAi) and klp-19/spd-1 (RNAi).* **B.** Plot of the centrosome to chromosome distance (half-spindle length, both halves combined) throughout anaphase for control embryos and embryos treated with *gpr-1/2 (RNAi), klp-19 (RNAi) and klp-19/gpr-1/2 (RNAi).* **C.** Bar plot of the chromosome to pole separation rate throughout anaphase for control embryos and embryos treated with RNAi as shown in A and B. **D.** Plot of the centrosome to chromosome distance (half-spindle length, both halves combined) throughout anaphase for control embryos and embryos treated with *zen-4 (RNAi), zen-4/ klp-19 (RNAi), klp-7 (RNAi)* and *klp-7/ klp-19 (RNAi).* **E.** Bar plot of the chromosome to pole separation rate throughout anaphase for control embryos and embryos treated with RNAi as shown in D. **F.** Bar plot of the chromosome to centrosome distance in metaphase and 60s after anaphase onset in the different conditions shown in A, B and D. **G.** Standard deviation of spindle pole-to-pole filtered distances reporting stability for *N* = 7 *klp-19(RNAi)* treated embryos versus *N* = 9 control, during early anaphase ranging from 10 to 72 s after anaphase onset and late anaphase (72 to 134 s). Errors bars are standard deviation.

However, when quantifying the spindle elongation and chromosome segregation in the C. elegans embryos after depletion of some of the midzone proteins we noticed that in some cases the distance between chromosomes and poles did change. Depletion of SPD-1 led to a noticeable reduction of the half-spindle length during anaphase. While the half spindle decreased by 0.5µm during anaphase in control, it shortened 1.25µm in *spd-1 (RNAi)* embryos (Fig. 8). Similarly, we observed a decrease in the distance between poles and chromosomes when depleting ZEN-4 as well as KLP-7, which both have been shown to lead to spindle breakage in anaphase (Fig. 8). These findings imply that in the absence of an intact spindle midzone the half-spindle length decreases throughout anaphase. In contrast, depletion of KLP-19 significantly reduced alterations in half-spindle length in comparison to control. In addition, co-depletion of KLP-19 effectively counteracted the reduction in pole-to-chromosome distance seen in embryos depleted of SPD-1, ZEN-4, and KLP-7. These observations suggest that a more robust spindle midzone, characterized by increased microtubule overlap, may enhance overall spindle stability. Interestingly this effect of KLP-19 was not observed with co-depletion of GPR-1/2 and thus removal of cortical forces (Fig 8). To further test this idea, we probed the pole-pole distance micro-fluctuations using the filtered standard deviation. We split the anaphase into early (from 10 to 72 s after anaphase onset) and late (72 to 134 s), as the spindle elongation mostly happened during the former. Upon *klp-19(RNAi)*, SD_l_ was reduced to 76 ± 3 % of the control value at early anaphase and to 83 ± 2 % at late anaphase (Fig 8G). This significant reduction in length fluctuations upon *klp-19(RNAi)* suggests that the spindle midzone may become more rigid during anaphase, coupling the two poles more strongly. This is consistent with increased microtubule overlap in the midzone and suggests a mechanical coupling within the spindle.

## Discussion

In this study we addressed the role of KLP-19 in the spindle midzone during anaphase in the *C. elegans* one-cell embryo. Numerous conserved proteins are associated with the midzone, coordinating its assembly and function. The microtubule cross-linker PRC1 plays a critical role in midzone stability in mammalian cells. In agreement with this, depletion of the *C. elegans* homolog, SPD-1, leads to spindle rupture during mitosis in response to pulling forces. Previous data has proposed that PRC1 and Kif4a are involved in the regulation of microtubule overlap in the spindle midzone. Our study demonstrates that the overlap of microtubules in the midzone remains constant during anaphase and is independent of pulling forces. We find that the size of the microtubule overlap is dependent on KLP-19 as the overlap increases upon *klp-19 (RNAi)*. Our data suggests that KLP-19 affects microtubule dynamics, most likely by stalling microtubule growth and promoting turn-over, which is in agreement with previously reported roles of the mammalian homolog KIF4a (Hu et al., 2011; Nunes Bastos et al., 2013; Subramanian et al., 2013). Loss of KLP-19 thus leads to increased microtubule growth and length in the midzone and with that an increase in microtubule interactions in *C. elegans*.

Аlthough previous publications suggested that PRC1 recruits Kif4a, we could not detect a decrease in KLP-19 signal in the midzone after depletion of SPD-1 in *C. elegans* embryos, suggesting that the localization of KLP-19 to the midzone is independent of SPD-1. Further analysis showed that the localization of KLP-19 to the midzone is dependent on BUB-1 as well as AIR-2. This is in agreement with previously published work showing that KLP-19 requires BUB-1 to localize between the bivalents in *C. elegans* meiosis (Dumont et al., 2010). As the localization of BUB-1 is dependent on AIR-2 (Dumont et al., 2010; Wignall & Villeneuve, 2009) it is likely that AIR-2 could prevent the localization of KLP-19 to the midzone by inhibiting the localization of BUB-1. However, in mammalian cells Aurora B phosphorylation of KIF4A has been shown to activate its microtubule-stimulated ATPase activity and enhances KIF4A binding to PRC1 and the antiparallel microtubule overlap (Douglas & Mishima, 2010; Nunes Bastos et al., 2013) suggesting that there could also be a direct interaction of KLP-19 with AIR-2. Further investigations would be required to determine the mechanism of KLP-19 translocation to the spindle midzone.

KLP-19 has been shown to play an important role during chromosome congression and the generation of polar ejection forces (Powers et al., 2004). Thus, KLP-19 has been described as a chromokinesin, sliding chromosomes towards the plus-end of microtubules. Our study confirmed the role of KLP-19 during congression. However, while we can not exclude that KLP-19 actively transports chromosomes, based on our data and KLP-19’s effect on microtubule dynamics, it is also possible that chromosome bound KLP-19 directly affects microtubule dynamics at the chromosomes. Regulating microtubule dynamics could then affect the forces that microtubules generate on chromosomes during congression, thus contributing to chromosome alignment. Therefore, it would be interesting to determine the detailed role of KLP-19 during congression in the future.

Our study reveals unexpected findings regarding the role of KLP-19 in spindle mechanics and chromosome segregation. Depletion of KLP-19 surprisingly acts as a protective mechanism for the spindle midzone, rescuing it from the disruptive effects of various perturbations, including *spd-1 (RNAi), zen-4 (RNAi), klp-7 (RNAi),* and GPR-1/2 overexpression. This protection is linked to the regulation of microtubule dynamics within the midzone, resulting in the formation of longer and more stable microtubules that likely engage in increased interactions. These changes in microtubule behavior have broader implications for the organization of the entire midzone and, possibly the spindle itself, as we observed global alterations in microtubule polymerization dynamics.

Our observations align with recent studies demonstrating that, in spindles undergoing both anaphase A and B, chromosome motion mirrors that of midzone microtubules (Yu et al., 2019). Disruption of the midzone leads to a complete cessation of chromosome motion, indicating that midzone microtubules generate the pushing forces required for proper chromosome segregation. However, our data raises a crucial question: does the enhanced stability of the midzone, resulting from KLP-19 depletion, compromise the mechanics of chromosome segregation?

To address this question, we must consider the three primary sites of force generation within the spindle: pulling forces on astral microtubules, depolymerization of kinetochore microtubules, and forces originating within the spindle midzone. In normal spindles, these forces collectively contribute to spindle positioning and chromosome segregation. Previous research in *C. elegans* embryos suggests that chromosome segregation is predominantly driven by forces generated within the spindle midzone, with cortical pulling forces primarily responsible for spindle positioning. Forces generated by kinetochore microtubules play a minor role in chromosome segregation in this context.

Our data demonstrates that the removal of cortical pulling forces in *C. elegans* does not prevent chromosome segregation but significantly hinders poleward motion of spindle poles, leading to shorter anaphase spindles and a reduction in the pole-to-chromosome distance. Conversely, weakening the spindle midzone, achieved through SPD-1 or ZEN-4 depletion, results in rapid pole separation and an increase in chromosome segregation velocity. Notably, the pole-to-chromosome distance decreases when the integrity of the midzone is compromised. These findings suggest that, in *C. elegans* spindles, kinetochore microtubules may depolymerize during mitosis, but this process is typically counteracted by the stronger forces generated by the spindle midzone and astral microtubules. When the midzone or astral microtubules are impaired, there is insufficient resistance to these weaker forces, resulting in a decrease in the pole-to-chromosome distance.

Our data further indicates that the absence of KLP-19 results in a more robust and resilient midzone network, leading to reduced rates of chromosome and pole separation during mitosis. Intriguingly, the dynamics of pole and chromosome segregation rates after KLP-19 depletion closely resemble those observed after the removal of cortical pulling forces. Additionally, the pole-to-chromosome distance remains remarkably stable in KLP-19-depleted embryos throughout anaphase. These findings suggest that enhanced midzone stability impacts the transmission of pulling forces within the spindle. Consistently, the stability of the pole-pole distance over time is increased during metaphase. Co-depletion of GPR-1/2 and KLP-19 does not further affect chromosome segregation but primarily influences pole separation, leading to a decrease in spindle elongation and a reduction in the pole-to-chromosome distance due to the loss of cortical pulling forces.

The significance of the midzone in chromosome segregation is underscored by our results. Alterations in microtubule dynamics, stability, length, and interactions within the midzone result in a corresponding shift in the overall network organization. While this reconfigured network becomes more resistant to pulling forces, it can also interfere with the rates, fidelity, and quality of chromosome segregation. These findings emphasize the necessity for the midzone network to maintain its dynamic nature and remain connected to the broader spindle machinery.

Our ultrastructural data hints at a potential connection between the midzone and the rest of the spindle through a specific subset of microtubules the inter-spindle microtubules (iSMTs), which span between the segregating chromosomes and the spindle poles (Fig. 1A). These connections likely facilitate force transmission, whether active or passive, among the midzone, astral microtubules, and the cortex during anaphase. Such interactions contribute to the stability of the spindle network. Any disruptions within the midzone, such as alterations in microtubule numbers and interactions, can have far-reaching effects on spindle structure and function.

While some of our findings regarding the role of KLP-19 during chromosome segregation may be specific to *C. elegans* due to strong cortical pulling forces, we propose that the regulation of microtubule length and interactions represents a critical mechanism for establishing a robust midzone organization essential for proper chromosome segregation. Moreover, our study suggests that KLP-19, in coordination with SPD-1, constitutes a conserved midzone module that dynamically interacts with the broader spindle machinery. Our data underscores how midzone organization influences spindle mechanics, particularly half-spindle length, and the spindle’s response to pulling forces.

In summary, the spindle midzone serves as a mechanical link between the two spindle halves, experiencing tension from cortical force generators crucial for spindle positioning and chromosome segregation by promoting spindle elongation. This mechanical balance between stability and adaptability is essential for ensuring the proper segregation of chromosomes, not only in *C. elegans* embryos but also in mammalian cells, which also experience pulling forces (Dujardin & Vallee, 2002; C. B. O’Connell & Wang, 2000). The midzone is a microtubule network held together by motor proteins and cross-linkers, forming dynamic, reversible bonds that allow for rearrangements and plasticity throughout anaphase. Stability within this network depends on the equilibrium between connection breakage and reformation. Additionally, the length of microtubules plays a pivotal role in midzone stability, with longer microtubules enhancing stability but potentially impeding chromosome segregation through increased interactions. Future research should aim to elucidate the precise mechanics governing midzone stability and adaptability, including the roles of cross-linkers, motors, and microtubule length.

## Materials and Methods

### CRISPR/Cas9 injections:

Klp19-GFP tagged line was created via CRISPR/Cas9 mediated genome engineering using the self-excising cassette (SEC) method to facilitate screening and the guide RNA targeting sequence 5’ CAAGCGAAAGAGTCGTCGAA 3’ (Dickinson et al., 2013, 2015). Targeting sequence was cloned into pDD162 plasmid by using NEB’s Q5 Site-Directed Mutagenesis Kit to insert the targeting sequence into our Cas9-sgRNA construct (Addgene #47549) by using forward primer 5’-(CAAGCGAAAGAGTCGTCGAA)GTTTTAGAGCTAGAAATAGCAAGT-3’, and reverse primer 5’-CAAGACATCTCGCAATAGG-3’. The repair templates were generated by first cloning homology arms, synthesized by PCR using genomic DNA as a template, into plasmid: pDD282 with C-terminal GFP Tag with FLAG and flexible linker. Worm injections and transgenics selection was done according to the above cited protocol. The strain SR52 was created, expressing endogenously labeled KLP-19::GFP

### C. elegans strains

MAS91 (unc-119(ed3) III; ItIs37[pAA64; pie-1::mCherry::HIS58]; ruIs57[pie-1::GFP:: β-tubulin+unc-119(+)]) was used for experiments of fluorescence imaging, laser ablation, and fluorescence recovery after photobleaching. MAS91 was a gift from Martin Srayko.

ANA72 (adeIs1 [mex-5::spd-1::GFP + unc-119(+)] II. ltIs37 [pie-1p::mCherry::his-58 + unc-119(+)] IV. ) was a gift from Marie Delattre. ANA72 was used to quantify the localization of SPD-1.

*wow47 [ebp-2::gfp::3xflag)] II; zif-1(gk117) III; wyEx9745]* was a gift from Kang Shen. OD305 (*unc-119(ed3) III; ltIs138 [pJD14; pie-1/KNL-1::GFP ; unc-119 (+)], unc- 119(ed3) III; ltIs37 [pAA64; pie-1/mCherry::his-58; unc-119 (+)] IV)* was a gift from Karen Oegema and Arshad Desai.

The SPD-1 temperature sensitive mutant WH12 (SPD-1 (oj5)) (CGC) was crossed with JA1559 (*weIs21 [pJA138 (pie-1::mCherry::tub::pie-1)]; unc-119(ed3) III)* to generate strain SR58. TH384 (unc-119(ed3) III; ddIs79[mCherry::gpr-1(synthetic, CAI 1.0, artificial introns) unc-119(+)] ) was used for GPR-1/2 overexpression. Wild-type (N2) C. elegans embryos were used for the preparation of electron tomography.

Oscillations and filtered SD upon *klp-19(RNAi)* used strain JEP97 (genotype: unc-119(ed3) III; ltIs37 [pAA64; pie-1/mCHERRY::his-58; unc-119 (+)]IV;ddIs44[WRM0614cB02 GLCherry::tbg-1;unc-119(+)] ) was obtained by successive crossings involving in particular TH169 and OD1209 (Moyle et al., 2014; Woodruff et al., 2015). Spindle positioning micro-fluctuations analysis upon *klp-7(RNAi)* used strain TH65 (Srayko et al. 2005). The strain JEP97 was maintained at 20°C, while TH65 at 25°C, and both fed on OP50 bacteria on nematode growth medium plates.

All strains were maintained at 16°C and fed on OP50 bacteria on nematode growth medium plates (Brenner, 1974).

### RNAi

RNAi experiments were performed by feeding (Timmons & Fire, 1998). RNAi feeding was performed on NGM plates containing 50 mg/mL ampicillin and 1 mM IPTG (Isopropyl b-D-thiogalactopyranoside) and seeded with the HT115(DE3) bacterial strain containing the desired target sequence.

Feeding clones for GPR-1/2, SPD-1, KLP-7 and KLP-19 were obtained from the RNAi library (Fraser et al., 2000). L4 larvae were transferred to plates seeded with bacteria producing dsRNA and grown for 48 hr at 25C prior to analysis. Plasmid pMD082 (a gift from the Marie Delattre lab) containing the sequence of *zen-4* was cloned into the L4440 plasmid, which was transformed into HT115 bacteria to generate feeding clones for ZEN-4.

The feeding clone for AIR-2 (B0207.4) was purchased from Horizon Discovery. Feeding clones for BUB-1 (CUUkp3300H113Q), HCP-3 (DFCIp3320C1010038D), HCP-4 (CUUkp3300L171Q) and NDC-80 (DFClp3320H0110028D) were purchased from Source Bioscience. The Feeding clone for ZYG-1 was a gift from Kevin O’Connell (K. F. O’Connell et al., 2001).

For the oscillation experiments *klp-19(RNAi)* was performed by feeding for 96 hours on NGM plates with 4 mM IPTG in the agar, while *klp-7* used 3mM during 48h, as described previously (Kamath & Ahringer, 2003; Timmons & Fire, 1998). Imaging was performed at 23°C.

### Spinning disk confocal fluorescence imaging

Embryos for live-imaging experiments were obtained by dissecting gravid adult hermaphrodites in M9 buffer (42 mM Na_2_HPO4, 22 mM KH_2_PO_4_, 86 mM NaCl, and 1 mM MgSO_4_). One-cell embryos were mounted on slides with 2% agarose pad, overlaid with a 22 × 22-mm coverslip, and imaged at room temperature on a widefield fluorescence microscope (ECLIPSE Ti2; Nikon) equipped with a CFI Apo TIRF 60× 1.45 NA oil immersion lens and a CCD camera (iXon 897 Ultra EMCCDs) or on a 3i VIVO spinning-disc confocal microscope (Axio Examiner.Z1; Zeiss) equipped with Zeiss Plan-Apochromat 63x/1.40 oil microscope objective, 6 laser lines and a Hamamatsu ORCA-Flash4.0 scientific CMOS camera (Hamamatsu) for detection. The microscope was controlled by Nikon NIS-Elements software (Nikon). Imaging was initiated in one-cell embryos before pronuclear meeting and was terminated 3 min after anaphase onset. EBP-2::GFP movies acquired with a spinning-disc confocal microscope. Image processing was done with ImageJ Software (images acquired at 400 ms intervals). Acquisition parameters were controlled using a Slidebook 6.0 program (3i - Intelligent Imaging).

### Widefield Imaging for Oscillations.

Embryos were observed at the spindle plane at 23°C using a Zeiss Axio Imager upright microscope (Zeiss, Oberkochen, Germany) modified for long-term time-lapse. First, extra anti-heat and ultra-violet filters were added to the mercury lamp light path. Secondly, to decrease the bleaching and obtain optimal excitation, we used an enhanced transmission 12-nm bandpass excitation filter centred on 485 nm (AHF analysentechnik, Tübingen, Germany). We used a Plan Apochromat 100/1.45 NA (numerical aperture) oil objective. Images were acquired with an Andor iXon3 EMCCD (electron multiplying charge-coupled device) 512 × 512 camera (Andor, Belfast, Northern Ireland) at 33 frames per second and using Solis software.

### Centrosome tracking and fluctuation analysis

The tracking of labelled centrosomes and analysis of trajectories were performed by a custom tracking software developed using Matlab (The Mathworks) (Pecreaux et al., 2006; Pécréaux et al., 2016). Tracking of −20°C methanol-fixed γ-tubulin labelled embryos indicated accuracy to 10 nm. We bandpass filtered the time traces between 0.1 and 1.1 Hz using the robust local regression algorithm to remove mean, residual drift, avoid any contribution from spindle elongation and remove high-frequency noise due to the tracking algorithm. The standard deviation of this filtered track corresponded to the filtered standard deviation (Pécréaux et al., 2016).

### Laser ablation and FRAP

The FRAP experiment was conducted using a Yokogawa CSU-W1 SoRa dual cam spinning disk confocal. The microscope is equipped with an Acal BFi UV Opti-Microscan point scanner that we used for the FRAP experiment. This system is integrated with Nikon NIS Elements software for seamless experimental setup and data acquisition. The movies were acquired with a 60 × 1.27 NA water objective and 2.8× SoRa magnifier with 100 ms exposure times and 250 ms intervals (4 frames/s).

For laser ablation the intensity of the ablation laser was increased to achieve severing of the spindles.

FRAP was calculated with a combination of Fiji (Schindelin et al. 2012) and MATLAB (MATLAB and Statistics Toolbox Release 2012, The MathWorks, Nitick, USA). Time-lapse images of spindles expressing GFP:: β-tubulin and mCherry::histone (corresponding to chromosomes) were realigned in a routine for matching, rotation, and translation using Rigid Body of Fiji’s StackReg plug-in, so that the random displacement of the spindle due to the spontaneous motion of the worm was corrected.

### Sample preparation for electron tomography

Wild-type (N2) *C. elegans* embryos were collected in cellulose capillary tubes (Pelletier et al., 2006) and high-pressure frozen as described using an EM ICE high-pressure freezer (Leica Microsystems, Vienna, Austria) (Muller‑Reichert et al., 2007). Freeze substitution was performed over 2–3 d at −90°C in anhydrous acetone containing 1% OsO_4_ and 0.1% uranyl acetate. Samples were embedded in Epon/Araldite and polymerized for 2 d at 60°C. Serial semi thick sections (200 nm) were cut using an Ultracut UCT Microtome (Leica Microsystems, Vienna, Austria) collected on Pioloform-coated copper slot grids and post stained with 2% uranyl acetate in 70% methanol followed by Reynold’s lead citrate. For dual-axis electron tomography (Mastronarde 1997), 15 nm colloidal gold particles (Sigma-Aldrich) were attached to both sides of semi-thick sections to serve as fiducial markers for subsequent image alignment. A series of tilted views were recorded using an F20 electron microscopy (Thermo-Fisher, formerly FEI) operating at 200 kV at magnifications ranging from 5000× to 6500× and recorded on a Gatan US4000 (4000 px × 4000 px) CCD or a Tietz TVIPS XF416 camera. Images were captured every 1.0° over a ±60° range.

### Quantification of electron tomography data

We used the IMOD software package (http://bio3d.colorado.edu/imod) for the calculation of electron tomograms (Kremer et al., 1996). We applied the Amira software package for the segmentation and automatic tracing of microtubules (Stalling et al., 2005). For this, we used an extension to the filament editor of the Amira visualization and data analysis software (Redemann et al., 2014, 2017; Weber et al., 2012). We also used the Amira software to stitch the obtained 3D models in *z* to create full volumes of the recorded spindles (Redemann et al., 2017; Weber et al., 2012). The automatic segmentation of the spindle microtubules was followed by a visual inspection of the traced microtubules within the tomograms. Correction of the individual microtubule tracings included manual tracing of undetected microtubules, connection of microtubules from section to section, and deletions of tracing artifacts (e.g., membranes of vesicles).

### Data analysis for tomography

Data analysis was performed using the Amira software (Visualization Sciences Group, Bordeaux, France).

#### Length distribution of microtubules

For the analysis of the microtubules length distributions, we previously found that removing MTs that leave the tomographic volume only had minor effects on the length distribution (Redemann et al., 2017). Therefore, we quantified all microtubules contained in the volume. In addition, in all analyses, microtubules shorter than 100 nm were excluded to reduce errors due to the minimal tracing length.

#### Interaction analysis

For the detection of possible interactions in 3D, the Interaction and Distance modules of the SpindleAnalysis Toolbox were used in Amira.

Interactions computes possible microtubule intersections. An intersection is defined as a part of a microtubule that is closer to another microtubule than given a threshold (here 100nm). In detail, for each point of a microtubule, the distance to another microtubule is computed. Based on these distances, all parts of the microtubule are detected that lie closer to the other microtubule than the threshold. For each part an interaction is generated and stored in a spread sheet. For each interaction the approximated angle and the interaction start, end and length is sored, In addition, we defined the maximum angle of 90 and the minimal interaction length of 100nm to filter the interactions.

The distance module computes the distance between microtubules. For each point of a microtubule the closest segment of all other microtubules is detected and stored as a floating point attribute. Furthermore, for each microtubule, the closest other microtubule is detected. The distance between two microtubules is defined as the minimal distance between the segments. The ID and the distance are stored as attributes on the edges.

For each line segment of a microtubule the distance to the surrounding microtubules was computed analytically. The distance of a microtubule was defined as the minimum of all segment distances. Second, for each pair of microtubules the distance and the angle were computed. The distance between two microtubules was defined as the minimum of the distances between all their line segments. A 3D grid data structure was used to accelerate these computations. To reduce errors due to local distortions of the microtubules, the angle is defined by the angle between the lines through the start and end points of the microtubules. Third, based on these data an abstract graph was constructed, where each microtubule is represented as a vertex and each interaction (based on thresholds for interaction distance and angle) as an edge.

### Quantitative analysis in of Microtubule growth in *C. elegans* mitotic spindles.

EB-2 GFP normalized mean intensity was measured in a fixed-size ROI in the midzone region. EB-2 GFP binding events were counted in a similar way to Srayko et al., 2005. Kymographs were generated in Fiji based on the lines half-distance between anterior centrosome and cortex. Segment of 4.5µm and 10% thresholding was used to segment EB-2 binding events and count them. Spd1-GFP and EB2-GFP profiles were measured with a mean profile intensity of the ROI box across the spindle.

### Statistical analysis.

Statistics are presented as mean ± SEM, and *p* values were calculated by the “ttest2” function in MATLAB.

### Second harmonic generation imaging and two-photon florescence imaging

Simultaneous SHG imaging and TP imaging were constructed around an inverted microscope (Eclipse Ti, Nikon, Tokyo, Japan), with a Ti:sapphire pulsed laser (Mai-Tai, Spectra-Physics, Mountain View, CA) for excitation (850 nm wavelength, 80 MHz repetition rate, ∼70 fs pulse width), a commercial scanning system (DCS-120, Becker & Hickl, Berlin, Germany), and hybrid detectors (HPM-100-40, Becker & Hickl). The maximum scan rate of the DCS-120 is ∼2 frames/s for a 512 × 512 image. The excitation laser was collimated by a telescope to avoid power loss at the XY galvanometric mirror scanner and to fill the back aperture of a water-immersion objective (CFI Apo 40× WI, NA 1.25, Nikon). A half-wave plate (AHWP05M-980) and a quarter-wave plate (AQWP05M-980) were used in combination to achieve circular polarization at the focal plane, resulting in equal SHG of all orientations of microtubules in the plane, unbiased by the global rotation of the spindle, the spatial variation in the angle of the microtubules, and the local angular disorder of microtubules. Forward-propagating SHG was collected through an oil-immersion condenser (1.4, Nikon) with a 425/30 nm filter (FF01-425/30-25, Semrock). Two-photon fluorescence was imaged with a non-descanned detection scheme with an emission filter appropriate for green-fluorescent-protein (GFP)-labeled tubulin in *C. elegans* (FF01-520/5-25, Semrock, Rochester, NY).

Both pathways contained short-pass filters (FF01-650/SP-25, Semrock) to block the fundamental laser wavelength. Image analysis was performed with MATLAB (The MathWorks, Natick MA), and ImageJ (National Institutes of Health, Bethesda, MD.

### Mitotracker

Timelapse fluorescent image sequences were processed in Python using nd2reader (https://github.com/Open-Science-Tools/nd2reader), OpenCV (https://pypi.org/project/opencv-python/), and scipy (https://scipy.org) along with other standard packages for data analysis. Multi-channel images were converted to 8-bit per channel and then split by spindle and DNA channels. Embryos remained in a relative fixed position during observation. Outlines of individual embryos were determined by creating a median projection of low background fluorescence of the GFP microtubule signal along the time axis, followed by watershed segmentation. The embryo outlines were used to create masks to eliminate all fluorescent microtubule and chromatid signals external to the individual embryo. To identify the absolute position of spindle poles and chromatids, a gaussian blur with a 3×3 kernel was applied to each channel at each timepoint, followed by binary thresholding using the top 25% (191–255) and top 50% (127–255) signal intensity for spindle poles and chromatid, respectively. Positions of each spindle poles and chromatid groups were determined as center or edge of the binarized objects, respectively. The midzone was determined as the half-distance between both spindle poles. The distance and velocity of the edge of the leading chromatids (advancing toward each spindle pole) was plotted over time relative to this midzone position. The oscillation of each spindle pole was tracked and plotted independently relative to a fixed reference axis. By default, this reference axis corresponded to the long axis of the embryo; however, the axis could be adjusted to align with the spindle axis of any chosen timepoint.

Kymographs for spindle pole and chromatid movements were created by applying a rotation to each frame such that the spindle pole-to-spindle pole axis in each frame aligns with a horizontal reference line. Each line in the final kymograph reflects a sum intensity projection of 10 pixels along the y-axis (+/− 5 pixel above/below the reference axis) of each rotated image. Enhanced kymographs were created from the original kymographs by applying standard adaptive thresholding and skeletonization to visualize the trajectories of spindle pole centers and chromatids’ leading edges. The Python code is available here: https://github.com/uvarc/mitosisanalyzer. Tracked data was used to plot distances between centrosomes and chromosomes at a specific time or during anaphase. Segregation rates were calculated from the displacement during first 40s after the beginning of anaphase.

## Supplementary Material

Supplement 1 Supplementary Figure 1. Depletion of midzone components affects spindle dynamics.
**A.** Stills of embryos in control embryos and after different RNAi treatments at 0s, 40s and 80s after anaphase onset. Embryos are expressing β-tubulin::GFP and histone::mCherry. Scale bar is 10µm. **B.** Same as A but for additional conditions.
Supplementary Figure 2. Depletion of KLP-19 affects chromosome segregation.
**A.** Stills of control embryos and after different RNAi treatments at 60s after anaphase onset. Embryos are expressing β-tubulin::GFP and histone::mCherry. Bottom images show chromosomes at metaphase and 60s after anaphase onset **B.** Bar plot of the chromosome area at metaphase and 60s after anaphase onset in control embryos and after different RNAi treatments. **C**. Stills of control embryos and embryos after *klp-19 (RNAi)* expressing kinetochore marker KNL-1::GFP and mCherry histone. Scale bar is 10µm in A or 5µm in C.
Supplementary Figure 3. KLP-19 affects polar ejection forces.
**A.** Stills of embryos treated with *zyg-1 (RNAi)* to induce the formation of monopolar spindles in the 2-cell stages. Embryos are expressing EBP-2::GFP and mCherry::Histone. Top shows *zyg-1 (RNAi)* embryos, bottom *zyg-1/ klp-19 (RNAi)* treated embryos. **B**. Plot of the distance between centrosomes and metaphase plate in each of the 2-cells (AB and P) in control and *klp-19 (RNAi)* embryos.
Supplementary Figure 4. KLP-19 prevents spindle rupture in SPD-1 (oj5).
**A.** Left: top: Stills of spd-1 (oj5) embryos expressing β-tubulin::GFP at NEBD and middle: 180s after NEBD. Bottom: Corresponding kymograph, Right: top: Stills of spd-1 (oj5) *klp-19 (RNAi)* embryos expressing β-tubulin::GFP at NEBD and middle: 180s after NEBD. Bottom: Corresponding kymograph. **B.** Plot of the spindle length over time in spd-1 (oj5) (blue) and spd-1 (oj5) *klp-19 (RNAi)* (orange). Scale bar 10µm
Supplementary Figure 5. Depletion of KLP-19 affects spindle dynamics.
**A.** Kymographs of *C. elegans* one-cell embryos from metaphase to anaphase in control and after *zen-4, zen-4/klp-19, klp-7, and klp-19/klp-7 (RNAi)* obtained by light microscopy. Microtubules are labeled with GFP, histone with mCherry. Orange errors point to lagging chromosomes. Scale Bar 10µm. **B.** Bar plot of the spindle length at metaphase in control embryos and after different RNAi treatments. **C**. Bar plot of the spindle length 60s after anaphase onset in control embryos and after different RNAi treatments. **D**. Bar plot of the chromosome distance 60s after anaphase onset in control embryos and after different RNAi treatments. **E.** Plot of pole-to-pole distance throughout anaphase in control embryos (green), after *zen-4 (RNAi)* (turquoise), *klp-19/ zen-4 (RNAi)* (blue), *klp-7 (RNAi)* (red) and *klp-7/klp-19 (RNAi)* (maroon). **F.** Plot of chromosome distance throughout anaphase in control embryos (green), after *zen-4 (RNAi)* (turquoise), *klp-19/ zen-4 (RNAi)* (blue), *klp-7 (RNAi)* (red) and *klp-7/klp-19 (RNAi)* (maroon). **G.** Bar Plot of centrosome segregation rate throughout anaphase in control embryos and embryos after different RNAi treatments. **H.** Bar Plot of chromosome segregation rate throughout anaphase in control embryos and embryos after different RNAi treatments. Error Bars are sem, significant differences (t-test) are indicated by asterisk.
Supplementary Figure 6. Depletion of KLP-19 prevents spindle breakage in embryos overexpressing GPR-1/2.
**A.** Stills of embryos expressing codon adapted (CAI 1.0) GPR-1/2 tagged with YFP leading to the formation of excessive cortical pulling forces. Embryos on the left are control, on the right embryos are treated with *klp-19 (RNAi)*. **B.** Bar plot of the pole separation rate in gpr-1/2 CAI 1.0 embryos (purple) and gpr-1/2 CAI embryos (pink) treated with *klp-19 (RNAi).* Scale bar 10µm.
Supplementary Figure 7. KLP-19 depletion affects SPD-1 signal in ZEN-4 depleted embryos.
**A.** Stills of control embryos (top), embryos treated with *zen-4 (RNAi)* (middle) and *zen-4/ klp-19 (RNAi)* (bottom). Embryos are expressing histone::mCherry and SPD-1::GFP. **B.** Plot of the normalized SPD-1::GFP intensity along the spindle axis in control embryos and after *klp-19/ zen-4 (RNAi)* **C.** Bar plot of the SPD-1::GFP half -intensity width in control embryos and embryos after *klp-19/ zen-4 (RNAi).*
Supplementary Figure 8. KLP-19 localization to the spindle midzone depends on BUB-1 and AIR-2.
**A.** Stills of control embryos (top), embryos treated with different RNAi throughout anaphase in embryos expressing α-tubulin::mCherry and KLP-19::GFP. **B.** Bar plot of the normalized KLP-19 intensity in the spindle center at 20s before metaphase, metaphase and 80s after anaphase onset.
Supplementary Figure 9. KLP-19 depletion leads to increased microtubule length and interactions.
**A.** 3D tomographic reconstruction obtained by electron tomography of all control and *klp-19 (RNAi)* embryos showing iKMTs (top), cMTs (middle) and microtubule that are color coded according to the local nearest distance to a neighboring microtubule, with red being 25nm and white larger than 100nm. Scale bar is 1µm **B.** Table of all quantified parameters for each dataset.
