# Supplementary material for: Chromokinesin Klp-19 regulates microtubule overlap and dynamics during anaphase in C. elegans
Source: bioRxiv. 2024 Dec 31:2023.10.26.564275. Originally published 2023 Oct 26. Preprint. [Version 2] doi: 10.1101/2023.10.26.564275 (PMC10634869; doi:10.1101/2023.10.26.564275)
Supplement: Supplement 1 [file NIHPP2023.10.26.564275v2-supplement-1.pdf]

bioRxiv preprint doi: https://doi.org/10.1101/2023.10.26.564275; this version posted December 31, 2024. The copyright holder for this preprint (which was not certified by peer review) is the author/funder, who has granted bioRxiv a license to display the preprint in perpetuity. It is made available under aCC-BY 4.0 International license.

Supplementary Figure Legends

Supplementary Figure 1. Depletion of midzone components affects spindle dynamics.
A. Stills of embryos in control embryos and after different RNAi treatments at 0s, 40s and 80s after anaphase onset. Embryos are expressing β-tubulin::GFP and histone::mCherry. Scale bar is 10μm. **B.** Plot of pole-to-pole distance throughout anaphase in control embryos (green), after *spd-1 (RNAi)* (blue), *klp-19/ spd-1 (RNAi)* (black) and *spd-1/ gpr-1/2 (RNAi)* (grey). Timepoint "0" is defined as anaphase onset **C.** Plot of chromosome distance throughout anaphase in control embryos (green), after *spd-1 (RNAi)* (blue), *klp-19/ spd-1 (RNAi)* (black) and *spd-1/ gpr-1/2 (RNAi)* (grey). **D.** Plot of pole-to-pole distance throughout anaphase in control embryos (green), after *klp-19 (RNAi)* (purple), *gpr-1/2 (RNAi)* (orange) and *klp-19/ gpr-1/2 (RNAi)* (maroon). **E.** Plot of chromosome distance throughout anaphase in control embryos (green), after *klp-19 (RNAi)* (purple), *gpr-1/2 (RNAi)* (orange) and *klp-19/ gpr-1/2 (RNAi)* (maroon). Error Bars are sem.

Supplementary Figure 2. Depletion of KLP-19 affects chromosome segregation.
A. Stills of control embryos and after different RNAi treatments at 60s after anaphase onset. Embryos are expressing EBP2::GFP (green) and histone::mCherry (red). Bottom images show chromosomes at anaphase onset and 60s after anaphase onset **B.** Bar plot of the chromosome area at metaphase and 60s after anaphase onset in control embryos and after different RNAi treatments. **C**. Stills of control embryos and embryos after *klp-19 (RNAi)* expressing kinetochore marker KNL-1::GFP and mCherry histone . Scale bar is 10μm in A and 5μm in C. Error Bars are

bioRxiv preprint doi: https://doi.org/10.1101/2023.10.26.564275; this version posted December 31, 2024. The copyright holder for this preprint (which was not certified by peer review) is the author/funder, who has granted bioRxiv a license to display the preprint in perpetuity. It is made available under aCC-BY 4.0 International license.

sem. The significance of differences between control and RNAi conditions was determined by two-tailed Student's *t*-tests (****$P < 0.0001$).

Supplementary Figure 3. KLP-19 affects polar ejection forces.
A. Stills of embryos treated with *zyg-1 (RNAi)* to induce the formation of monopolar spindles in the 2-cell stages. Embryos are expressing EBP-2::GFP (green) and mCherry::Histone. (magenta) Top shows *zyg-1 (RNAi)* embryos, bottom *zyg-1/ klp-19 (RNAi)* treated embryos. Scale bar 10µm **B**. Plot of the distance between centrosomes and metaphase plate in each of the 2-cells (AB and P) in control and *klp-19 (RNAi)* embryos. Error Bars are sem. The significance of differences between control and RNAi conditions was determined by two-tailed Student's *t*-tests (****$P <$ 0.0001).

Supplementary Figure 4. KLP-19 depletion does not directly affect cortical pulling forces.
A. Stills of embryos before (top) and 10 s after (bottom) laser microsurgery severing the posterior centrosome from the spindle in control (left), *klp-19 (RNAi)* (middle) and *gpr-1/2 (RNAi)* (right) treated embryos. **B.** Plot of the velocity of the anterior (continuous line) and posterior (dashed line) centrosome after laser microsurgery in control (green), *gpr-1/2 (RNAi)* (orange) and *klp-19 (RNAi)* (purple) treated embryos. The significance of differences between control and RNAi conditions was determined by two-tailed Student's *t*-tests (****$P < 0.0001$). Error bars are sem, Scale bar is 10µm.

Supplementary Figure 5. Spindle dynamics in *hcp-6 (RNAi)* treated embryos.
A. Plot of the spindle length at NEBD, Anaphase onset and 60s after anaphase onset in control embryos and embryos depleted of KLP-19, KLP-19/ SPD-1, SPD-1, HCP-6 and HCP-6/ SPD-1. **B.** Plot of the chromosome distance at 60s after anaphase onset. **C.** Plot of the spindle length from NEBD (0) through metaphase and anaphase in control *hcp-6 (RNAi)* and *klp-19 (RNAi)* treated embryos. The average timepoint of metaphase is indicated by the shaded boxes. **D.** Plot of the spindle length from NEBD (0) through metaphase and anaphase in *spd-1 (RNAi), hcp-6/ spd-1 (RNAi)* and *spd-1/ klp-19 (RNAi)* treated embryos. The average timepoint of metaphase is indicated by the shaded boxes. **E.** Bar plot of the average time from NEBD to anaphase onset for control and RNAi treated embryos. **F.** Plot of SPD-1 GFP intensity along the spindle axis in control embryos (green) and embryos after HCP-6 depletion (yellow). Color code: control embryos = green, *klp-19 (RNAi)* = purple, *klp-19/ spd-1 (RNAi)* = grey, *spd-1 (RNAi)* = blue, *hcp-6 (RNAi)* = yellow, *hcp-6/ spd-1 (RNAi)* = light green. The significance of differences between control and RNAi conditions was determined by two-tailed Student's *t*-tests (****$P <$ 0.0001). Error bars are sem.

Supplementary Figure 6. KLP-19 affects microtubule overlap in the spindle midzone
A. Plot showing the normalized intensity of SPD-1 GFP along the spindle axis in control embryos (green), embryos depleted of KLP-19 (red) and embryos after *klp-19/ gpr-1/2 (RNAi)*

bioRxiv preprint doi: https://doi.org/10.1101/2023.10.26.564275; this version posted December 31, 2024. The copyright holder for this preprint (which was not certified by peer review) is the author/funder, who has granted bioRxiv a license to display the preprint in perpetuity. It is made available under aCC-BY 4.0 International license.

(blue). “0” is the spindle center **B.** Left: Two-photon microscopy images of β-tubulin::GFP in control (top), *klp-19/ gpr-1/2 (RNAi)* and klp-19/ spd-1 (RNAi) treated embryos. Right: Corresponding SHG images. Scale Bars are 10µm left, 5µm right images **C.** Plot of the polarity of microtubules along the spindle axis (0= spindle center) of control embryos and after *klp-19/ gpr-1/2 (RNAi)* and *klp-19/ spd-1 (RNAi)*. Error bars are sem.

Supplementary Figure 7. KLP-19 localization to the spindle midzone depends on BUB-1 and AIR-2.
A. Stills of control embryos (top), embryos treated with different RNAis throughout anaphase in embryos expressing α-tubulin::mCherry and KLP-19::GFP. **B.** Bar plot of the normalized KLP-19 intensity in the spindle center at 20s before metaphase, metaphase and 80s after anaphase onset. Color code: control embryos = green, *air-2 (RNAi)* = yellow, *bub-1 (RNAi)* = red, *hcp-3 (RNAi)* = light green, *hcp-4 (RNAi)* = blue, *ndc-80 (RNAi)* = brown. The significance of differences between control and RNAi conditions was determined by two-tailed Student’s *t*-tests (****$P < 0.0001$). Error bars are sem.

Supplementary Figure 8. KLP-19 molecules form homodimers
A. Histogram plot showing the velocity of KLP-19. **B.** Enlarged region highlighted from the left panel in yellow. **C** Histogram plot showing KLP-19 run length on microtubules.
D. TIRF difference image showing newly appeared KLP-19 molecules. **E**. Example intensity trace of the single particle highlighted in yellow, showing two photobleaching steps. **F**. Plot showing the frequency of different KLP-19 oligomer sizes. **G**. Plot showing KLP-19 velocity as a function of particle brightness.

Supplementary Figure 9. KLP-19 depletion leads to increased microtubule length and interactions.
A. 3D tomographic reconstruction obtained by electron tomography of all control and *klp-19 (RNAi)* embryos showing iKMTs (top), cMTs (middle) and microtubule that are color coded according to the local nearest distance to a neighboring microtubule, with red being 25nm and white larger than 100nm. Scale bar is 1µm. (Control 1 and 2 were previously shown in a different context [7] and have been adapted) **B.** Table of all quantified parameters for each dataset.

Movies

Movie 1. Related to Figure 2
Time-lapse movie of a *C. elegans* one-cell embryo expressing EBP2 GFP and cherry Histone. Interval 2 sec.

Movie 2. Related to Figure 2

bioRxiv preprint doi: https://doi.org/10.1101/2023.10.26.564275; this version posted December 31, 2024. The copyright holder for this preprint (which was not certified by peer review) is the author/funder, who has granted bioRxiv a license to display the preprint in perpetuity. It is made available under aCC-BY 4.0 International license.

Time-lapse movie of a *C. elegans* one-cell embryo expressing EBP2 GFP and cherry Histone after *spd-1 (RNAi)*. Interval 2 sec.

Movie 3. Related to Figure 2
Time-lapse movie of a *C. elegans* one-cell embryo expressing EBP2 GFP and cherry Histone after *spd-/ gpr-1/2 (RNAi)*. Interval 2 sec.

Movie 4. Related to Figure 2
Time-lapse movie of a *C. elegans* one-cell embryo expressing EBP2 GFP and cherry Histone after *klp-19 (RNAi)*. Interval 2 sec.

Movie 5. Related to Figure 2
Time-lapse movie of a *C. elegans* one-cell embryo expressing EBP2 GFP and cherry Histone after spd-1/ *klp-19 (RNAi)*. Interval 2 sec.

Movie 6. Related to Figure 4
Time-lapse movie of metaphase cell-derived KLP-19::GFP moving on pre-polymerized, fluorescently labeled microtubules. Interval 1 sec. Still from this movie are shown in Figure 4B, Example 1

Movie 7. Related to Figure 4
Time-lapse movie of anaphase cell-derived KLP-19::GFP moving on pre-polymerized, fluorescently labeled microtubules. Interval 1 sec. Still from this movie are shown in Figure 4B, Example 2.

Movie 8. Related to Figure 4
Time-lapse movie of anaphase cell-derived SPD-1::GFP binding and concentrating on region of dense and brighter tubulin signal. Interval 1 sec. Still from this movie are shown in Figure 4E

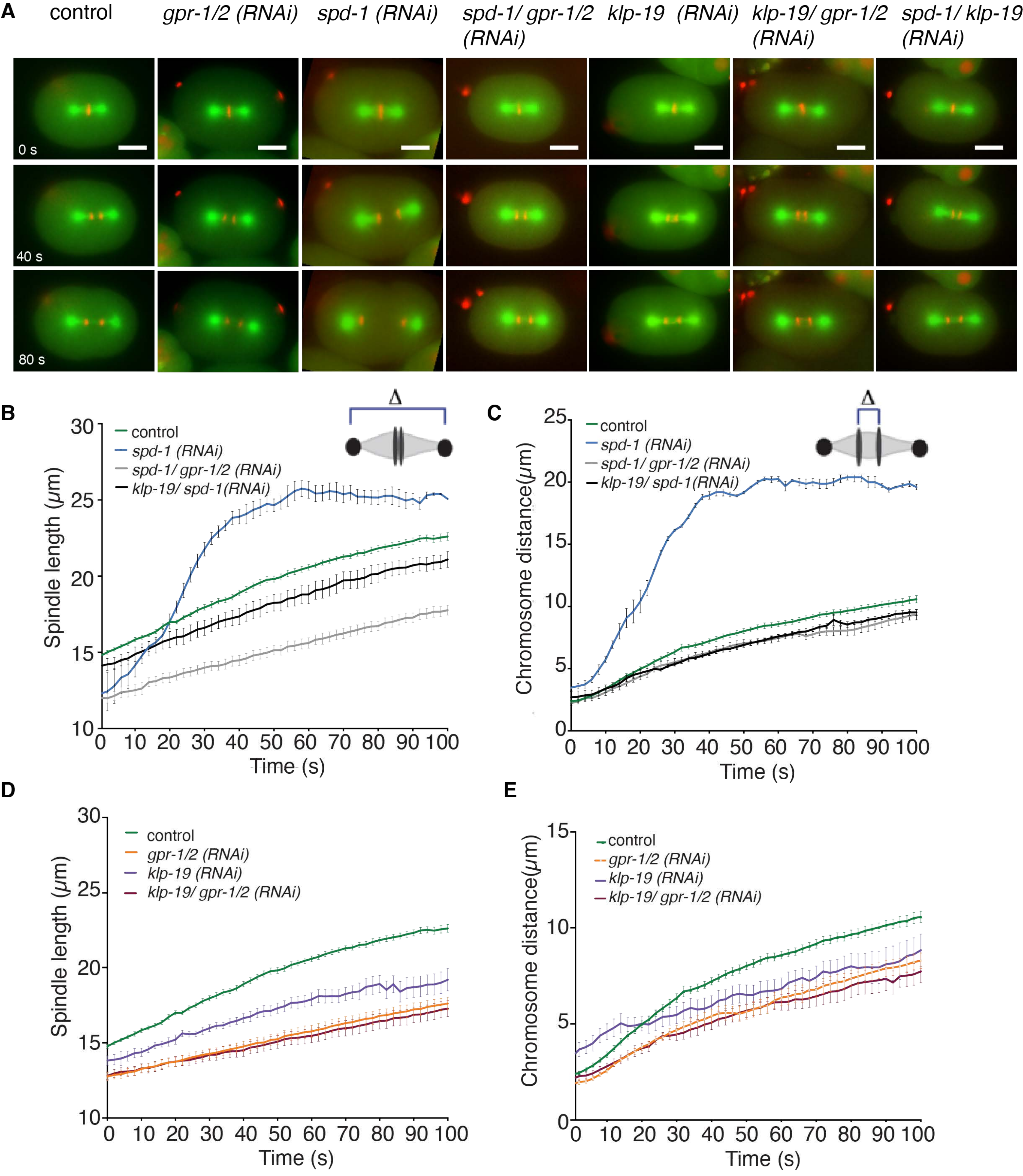
A
control
gpr-1/2 (RNAi)
spd-1 (RNAi)
spd-1/ gpr-1/2 (RNAi)
klp-19 (RNAi)
klp-19/ gpr-1/2 (RNAi)
spd-1/ klp-19 (RNAi)
0 s
40 s
80 s
B
Spindle length (μm)
Time (s)
control
spd-1 (RNAi)
spd-1/ gpr-1/2 (RNAi)
klp-19/ spd-1(RNAi)
Δ
C
Chromosome distance(μm)
Time (s)
control
spd-1 (RNAi)
spd-1/ gpr-1/2 (RNAi)
klp-19/ spd-1(RNAi)
Δ
D
Spindle length (μm)
Time (s)
control
gpr-1/2 (RNAi)
klp-19 (RNAi)
klp-19/ gpr-1/2 (RNAi)
E
Chromosome distance(μm)
Time (s)
control
gpr-1/2 (RNAi)
klp-19 (RNAi)
klp-19/ gpr-1/2 (RNAi)

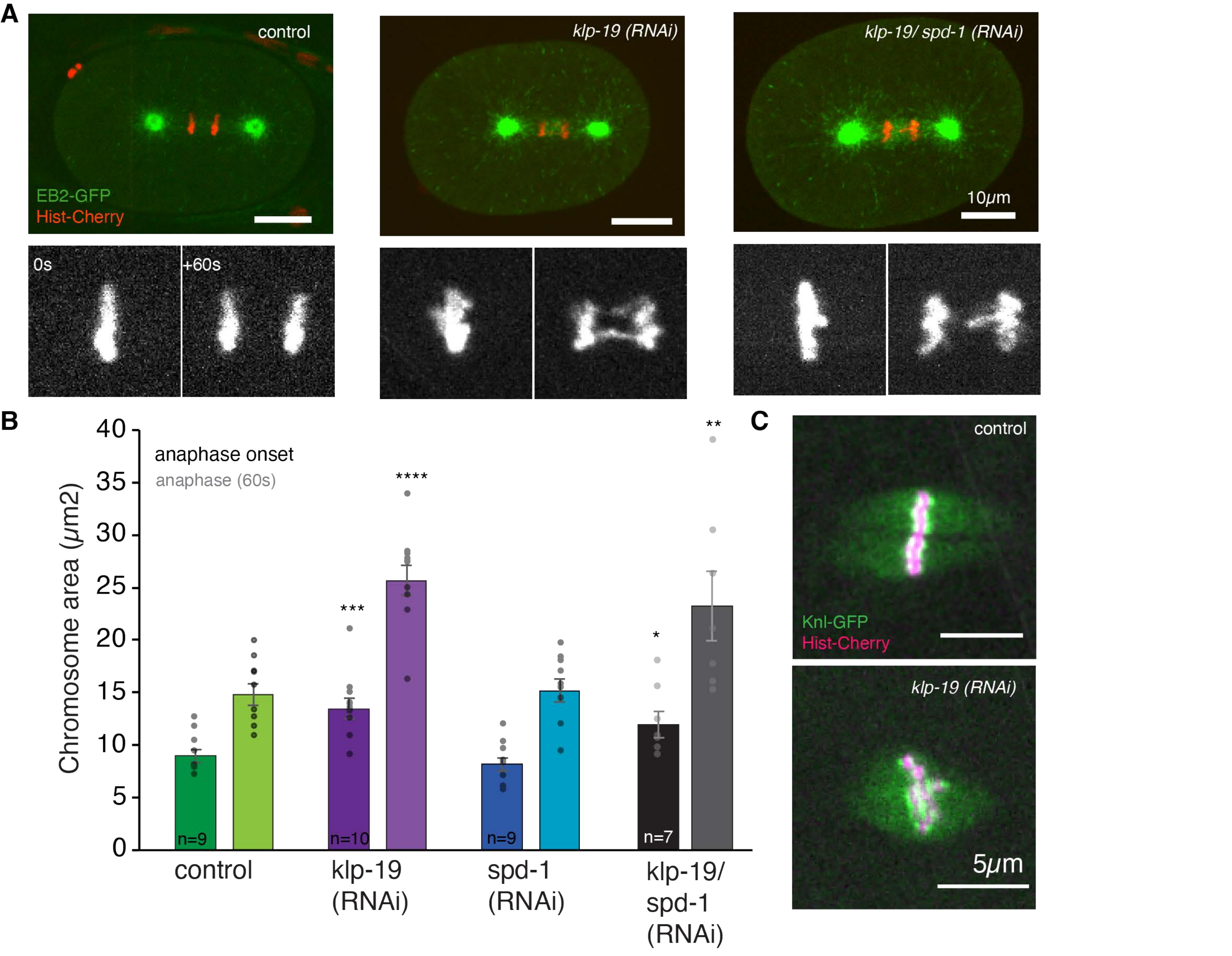

A
control
EB2-GFP
Hist-Cherry
klp-19 (RNAi)
klp-19/ spd-1 (RNAi)
10μm
0s
+60s
B
anaphase onset
anaphase (60s)
Chromosome area (μm2)
0
5
10
15
20
25
30
35
40
***
****
*
**
n=9
n=10
n=9
n=7
control
klp-19 (RNAi)
spd-1 (RNAi)
klp-19/ spd-1 (RNAi)
C
control
Knl-GFP
Hist-Cherry
klp-19 (RNAi)
5μm

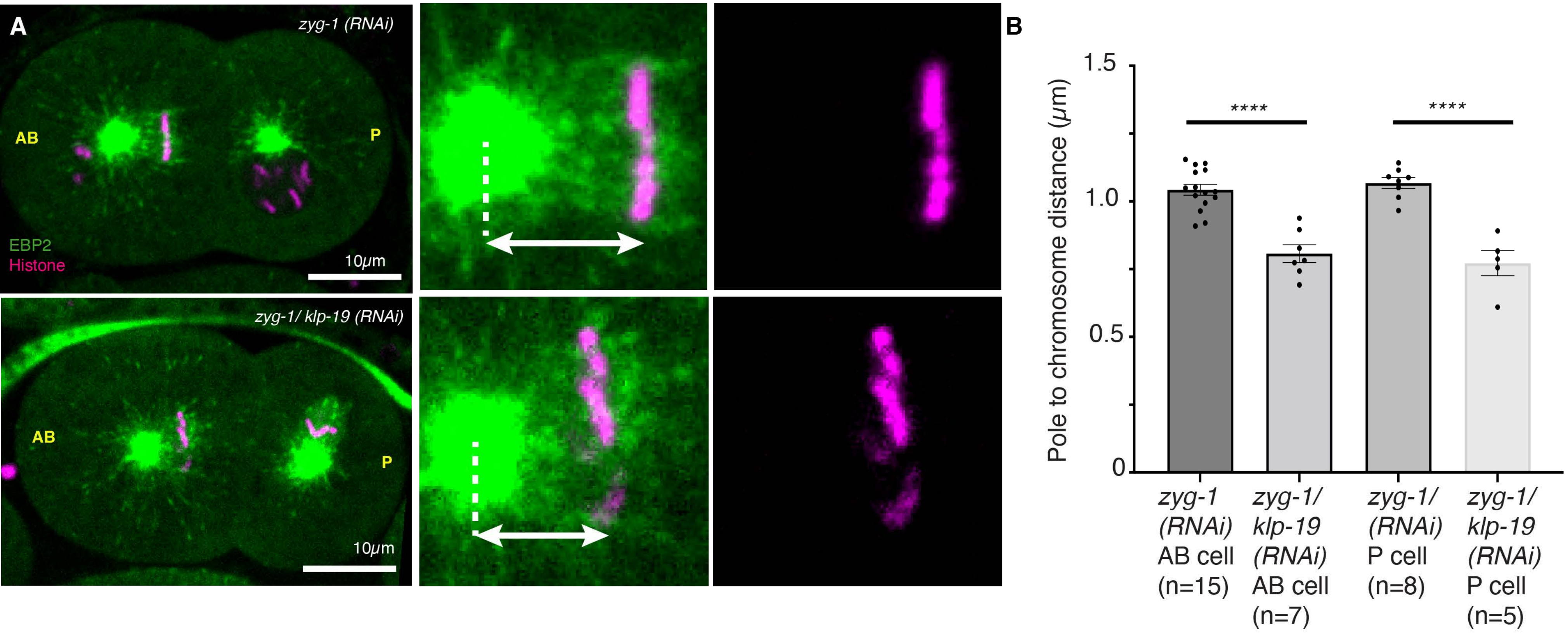
A
zyg-1 (RNAi)
AB
P
EBP2
Histone
10μm
zyg-1/ klp-19 (RNAi)
AB
P
10μm
B
1.5
1.0
0.5
0
Pole to chromosome distance (μm)
****
****
zyg-1 (RNAi) AB cell (n=15)
zyg-1/ klp-19 (RNAi) AB cell (n=7)
zyg-1/ (RNAi) P cell (n=8)
zyg-1/ klp-19 (RNAi) P cell (n=5)

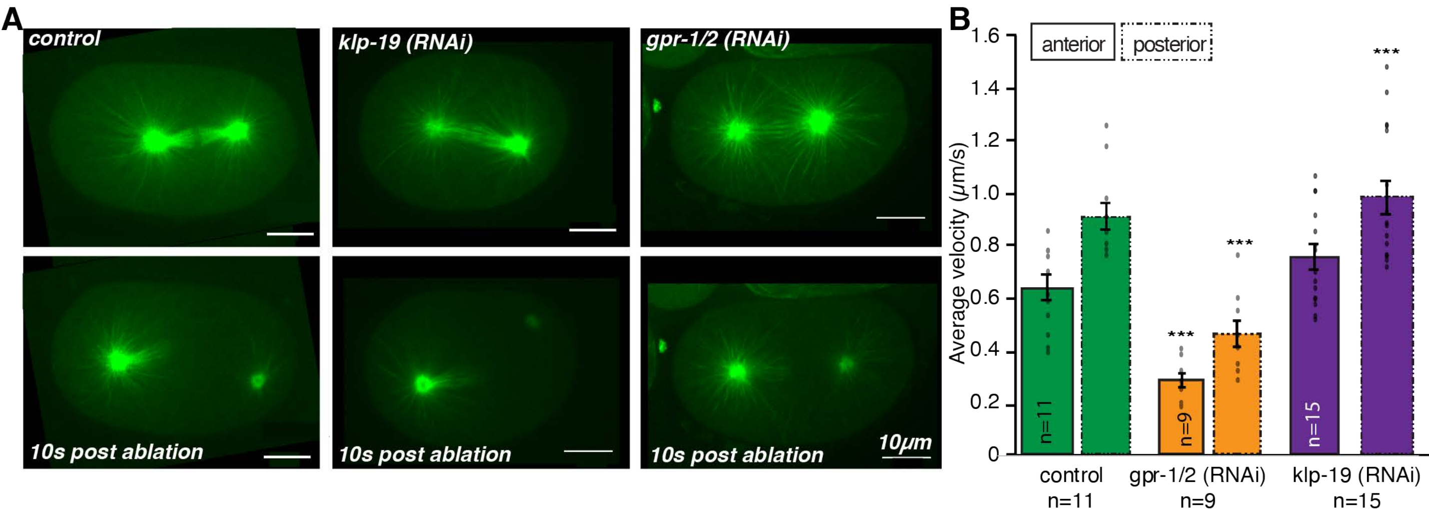

A
control
klp-19 (RNAi)
gpr-1/2 (RNAi)
10s post ablation
10s post ablation
10s post ablation
10μm
B
anterior
posterior
Average velocity (μm/s)
1.6
1.4
1.2
1.0
0.8
0.6
0.4
0.2
0
***
***
***
n=11
n=9
n=15
control
n=11
gpr-1/2 (RNAi)
n=9
klp-19 (RNAi)
n=15

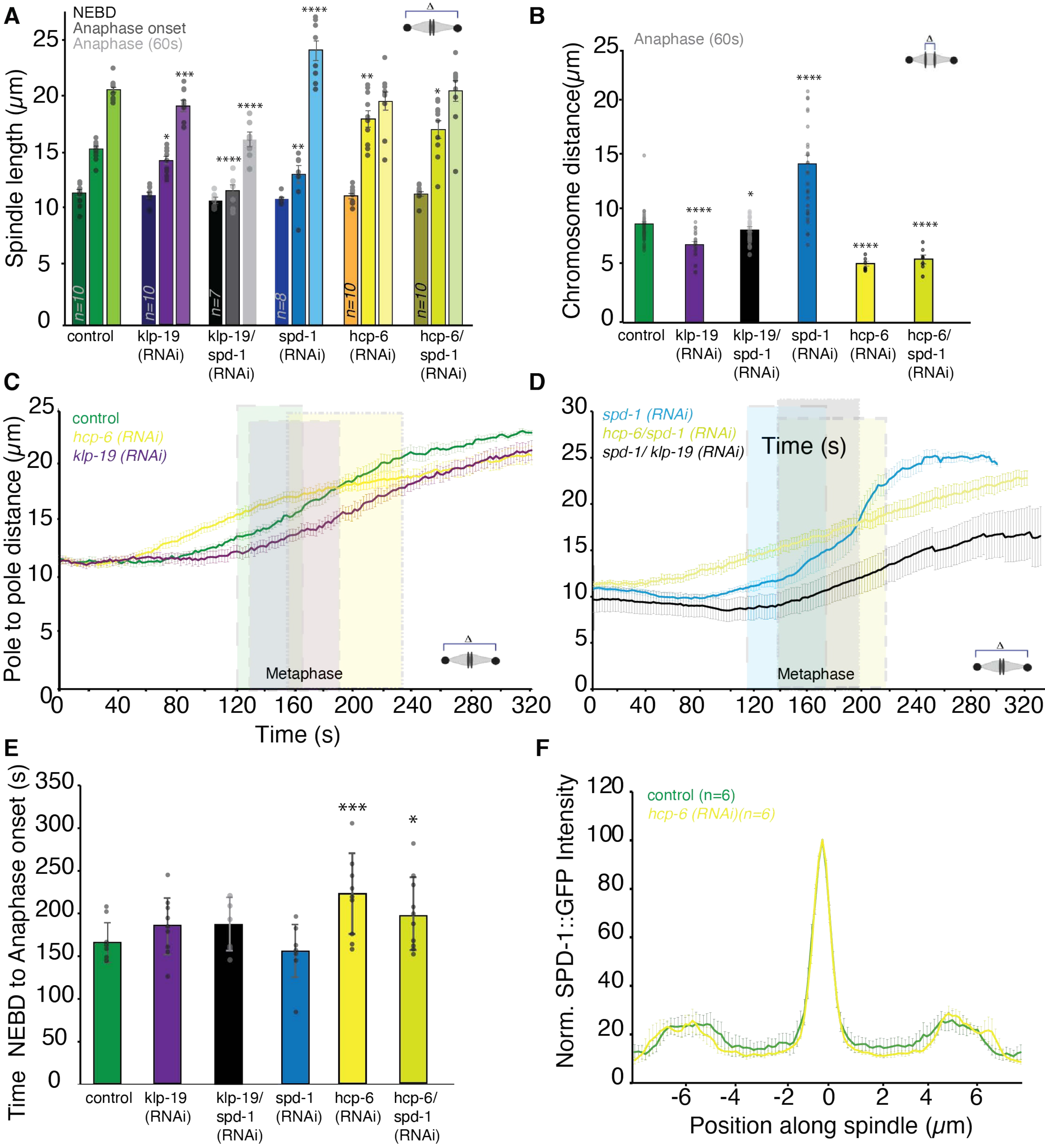
A
NEBD
Anaphase onset
Anaphase (60s)
Spindle length (μm)
0
5
10
15
20
25
n=10
n=10
n=7
n=8
n=10
n=10
*
***
****
****
**
****
**
*
control
klp-19 (RNAi)
klp-19/ spd-1 (RNAi)
spd-1 (RNAi)
hcp-6 (RNAi)
hcp-6/ spd-1 (RNAi)
Δ
B
Anaphase (60s)
Chromosome distance(μm)
0
5
10
15
20
25
****
*
****
****
****
control
klp-19 (RNAi)
klp-19/ spd-1 (RNAi)
spd-1 (RNAi)
hcp-6 (RNAi)
hcp-6/ spd-1 (RNAi)
C
control
hcp-6 (RNAi)
klp-19 (RNAi)
Pole to pole distance (μm)
0
5
10
15
20
25
Metaphase
0
40
80
120
160
200
240
280
320
Time (s)
D
spd-1 (RNAi)
hcp-6/spd-1 (RNAi)
spd-1/ klp-19 (RNAi)
Time (s)
0
5
10
15
20
25
30
Metaphase
0
40
80
120
160
200
240
280
320
E
Time NEBD to Anaphase onset (s)
0
50
100
150
200
250
300
350
***
*
control
klp-19 (RNAi)
klp-19/ spd-1 (RNAi)
spd-1 (RNAi)
hcp-6 (RNAi)
hcp-6/ spd-1 (RNAi)
F
control (n=6)
hcp-6 (RNAi)(n=6)
Norm. SPD-1::GFP Intensity
0
20
40
60
80
100
120
-6
-4
-2
0
2
4
6
Position along spindle (μm)

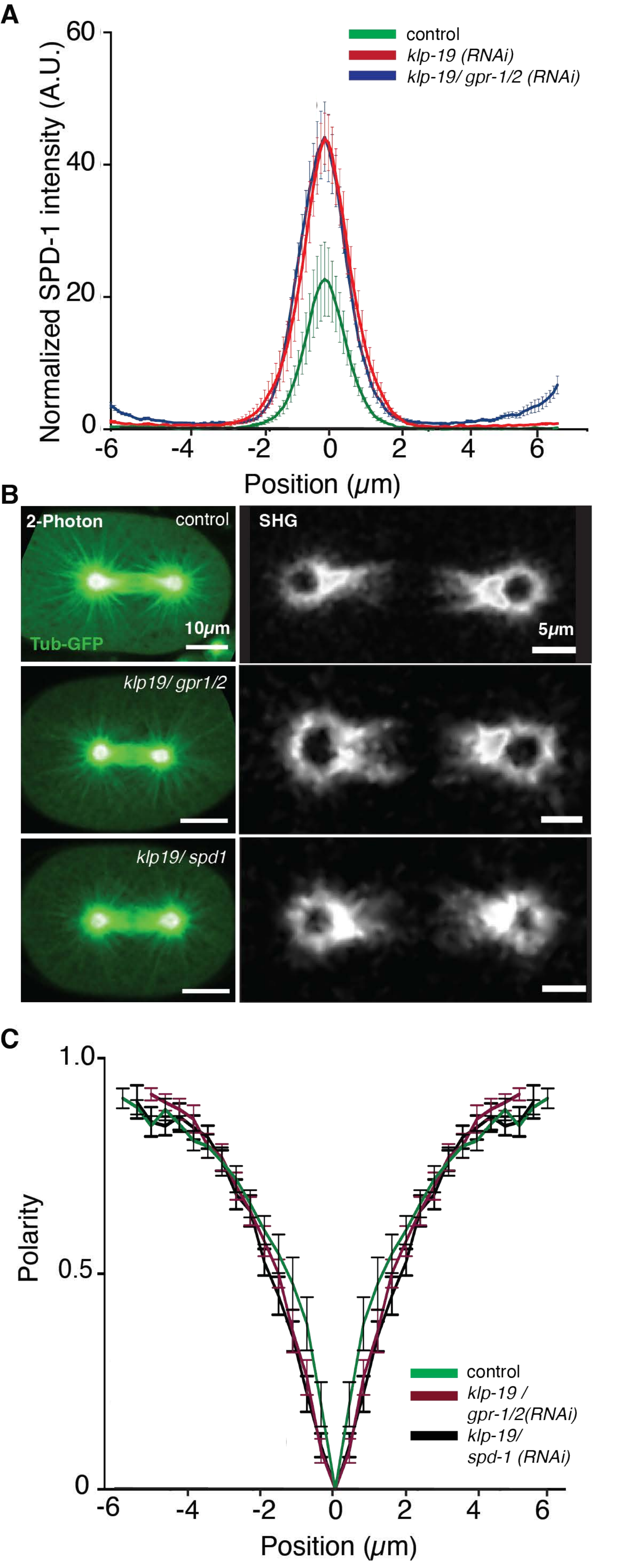
A
Normalized SPD-1 intensity (A.U.)
60
40
20
0
-6
-4
-2
0
2
4
6
Position (μm)
control
klp-19 (RNAi)
klp-19/ gpr-1/2 (RNAi)
B
2-Photon
control
Tub-GFP
10μm
SHG
5μm
klp19/ gpr1/2
klp19/ spd1
C
Polarity
1.0
0.5
0
-6
-4
-2
0
2
4
6
Position (μm)
control
klp-19 / gpr-1/2(RNAi)
klp-19/ spd-1 (RNAi)

bioRxiv preprint doi: https://doi.org/10.1101/2023.10.26.564275; this version posted December 31, 2024. The copyright holder for this preprint (which was not certified by peer review) is the author/funder, who has granted bioRxiv a license to display the preprint in perpetuity. It is made available under aCC-BY 4.0 International license.

A

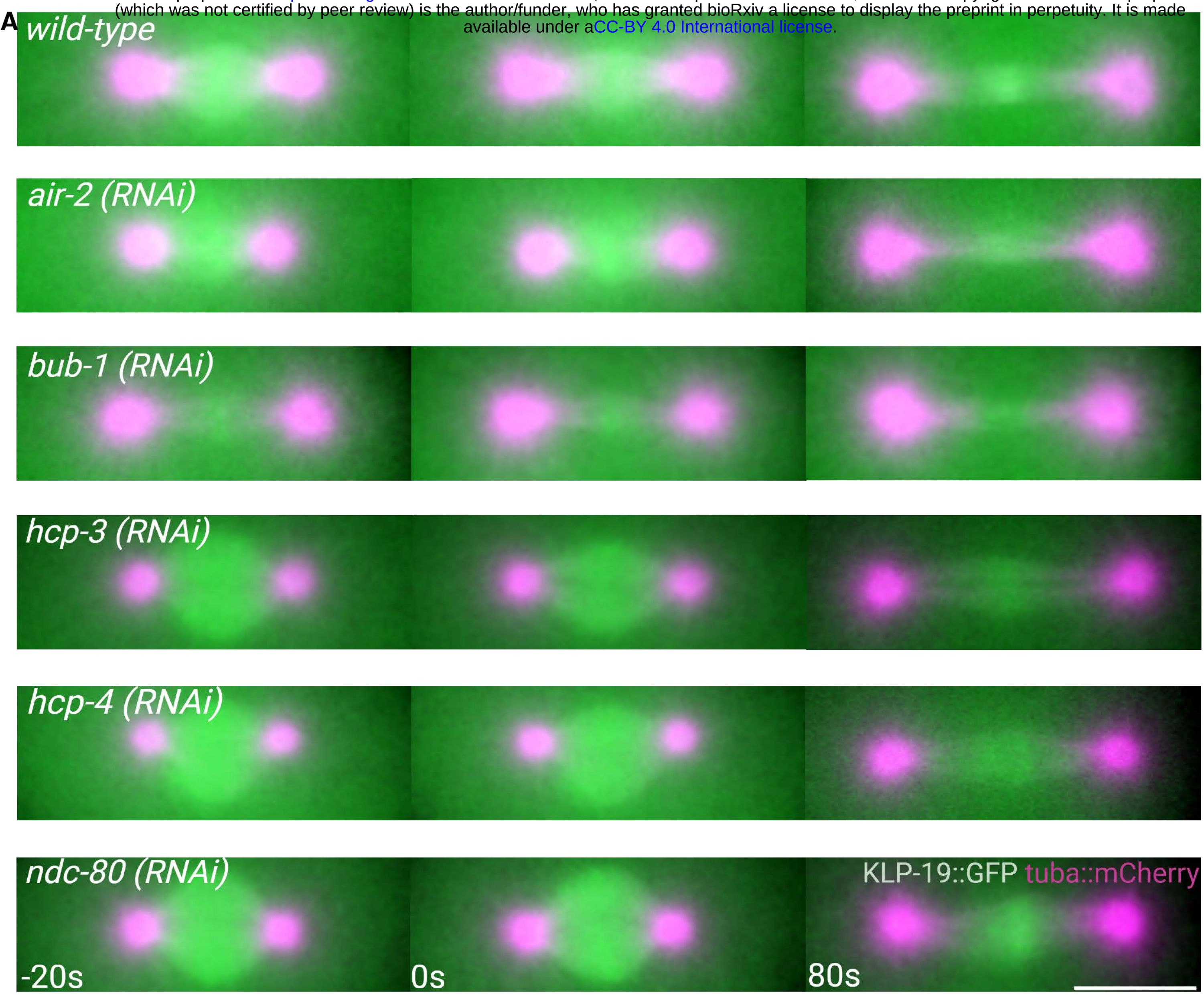

B

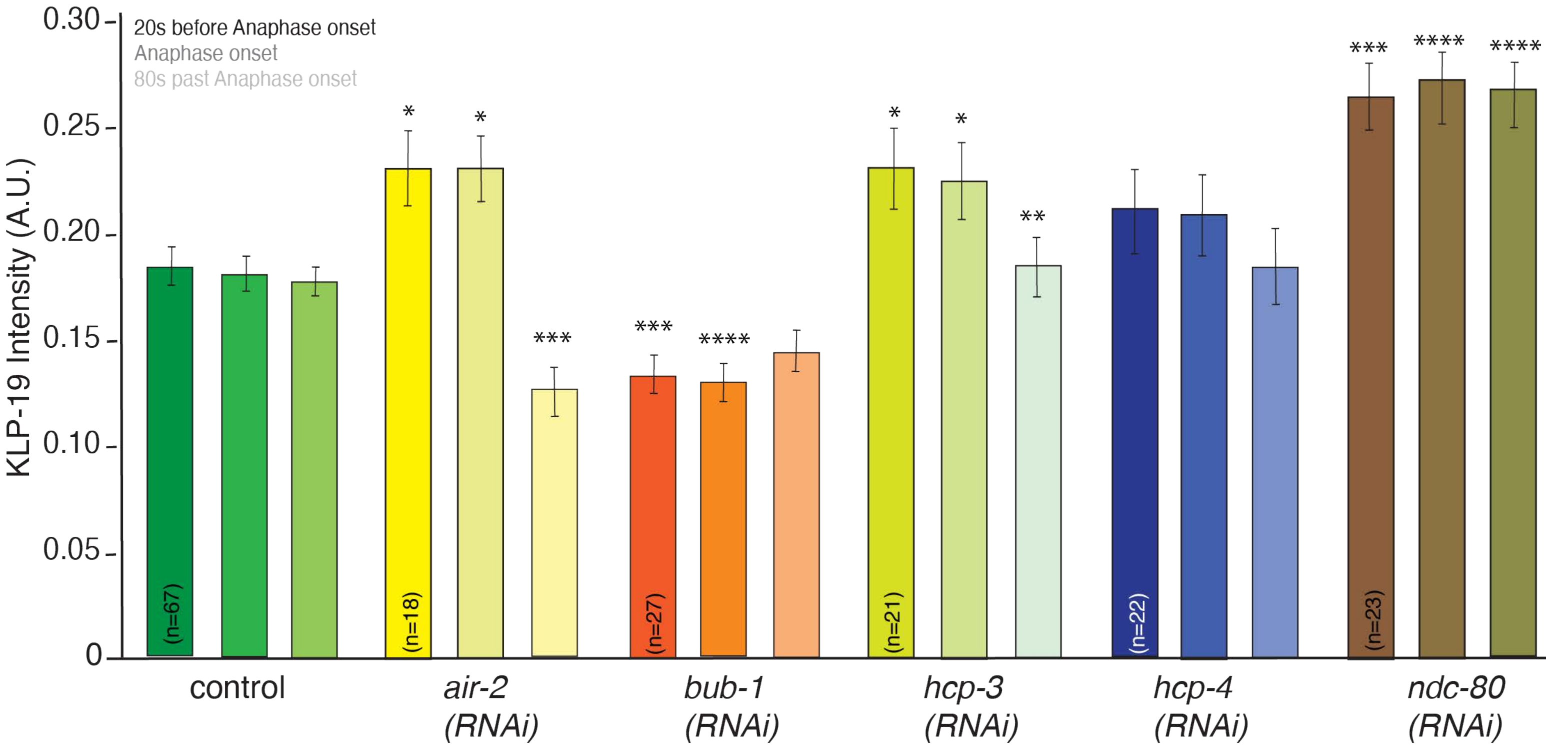

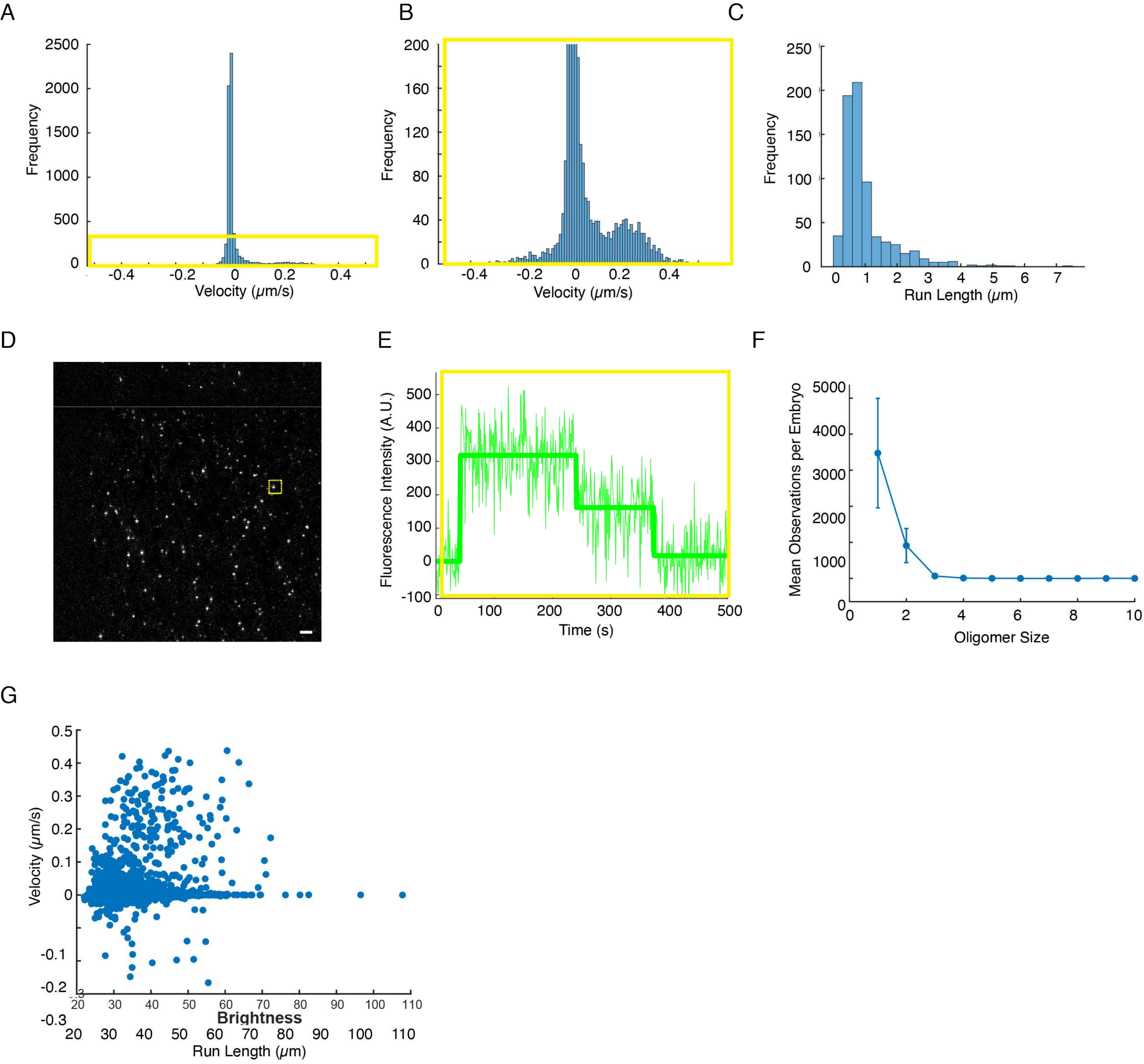
A
2500
2000
1500
1000
500
0
Frequency
-0.4
-0.2
0
0.2
0.4
Velocity (μm/s)
B
200
160
120
80
40
0
Frequency
-0.4
-0.2
0
0.2
0.4
Velocity (μm/s)
C
250
200
150
100
50
0
Frequency
0
1
2
3
4
5
6
7
Run Length (μm)
D
E
500
400
300
200
100
0
-100
Fluorescence Intensity (A.U.)
0
100
200
300
400
500
Time (s)
F
5000
4000
3000
2000
1000
0
Mean Observations per Embryo
0
2
4
6
8
10
Oligomer Size
G
0.5
0.4
0.3
0.2
0.1
0
-0.1
-0.2
-0.3
Velocity (μm/s)
20
30
40
50
60
70
80
90
100
110
Brightness
20
30
40
50
60
70
80
90
100
110
Run Length (μm)

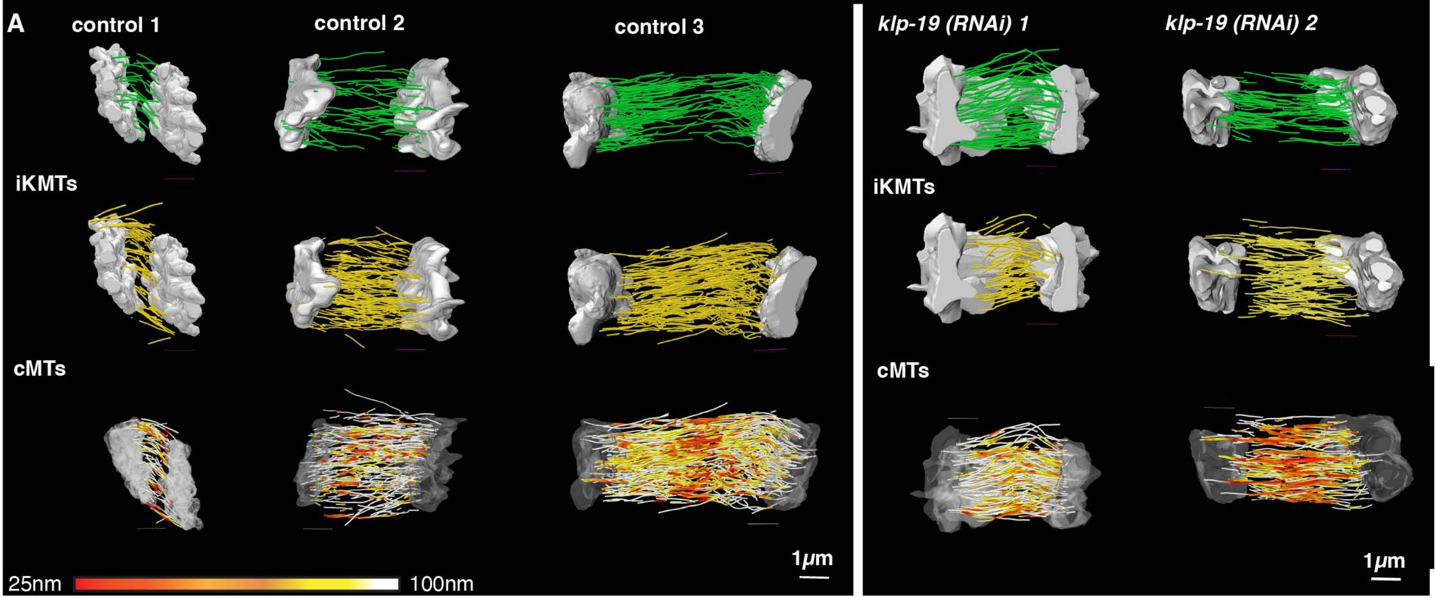

	Control 1	control 2	control 3	klp-19 (RNAi) 1	klp-19 (RNAi) 2
chrom dist μm	1.5	4	5.5	3	4.5
iKMT/μm^3	0.7	0.4	1.4	1.8	1.3
cMT/μm3	1.9	1.2	3.6	2.3	3
Av. MT Length μm	0.6 ± 0.03	0.95 ± 0.05	1.09 ± 0.04	1.12 ± 0.04	1.18 ± 0.05
AV. Interact. Length μm	0.39 ± 0.03	0.41 ± 0.01	0.46 ± 0.01	0.44 ± 0.01	0.56 ± 0.01
# Interact. per MT	0.4	1	3.3	3	1.6
Av. Interact. Distance nm	106 ± 6.6	83 ± 2.2	55 ± 1.3	62 ± 2.9	62 ± 2.3
