# Supplementary material for: Lack of chromokinesin Klp-19 creates a more rigid midzone and affects force transmission during anaphase in C. elegans
Source: bioRxiv. 2023 Oct 26:2023.10.26.564275. Preprint. [Version 1] doi: 10.1101/2023.10.26.564275 (PMC10634869; doi:10.1101/2023.10.26.564275)
Supplement: Supplementary file 1 [file media-1.pdf]

Supplementary figure 1

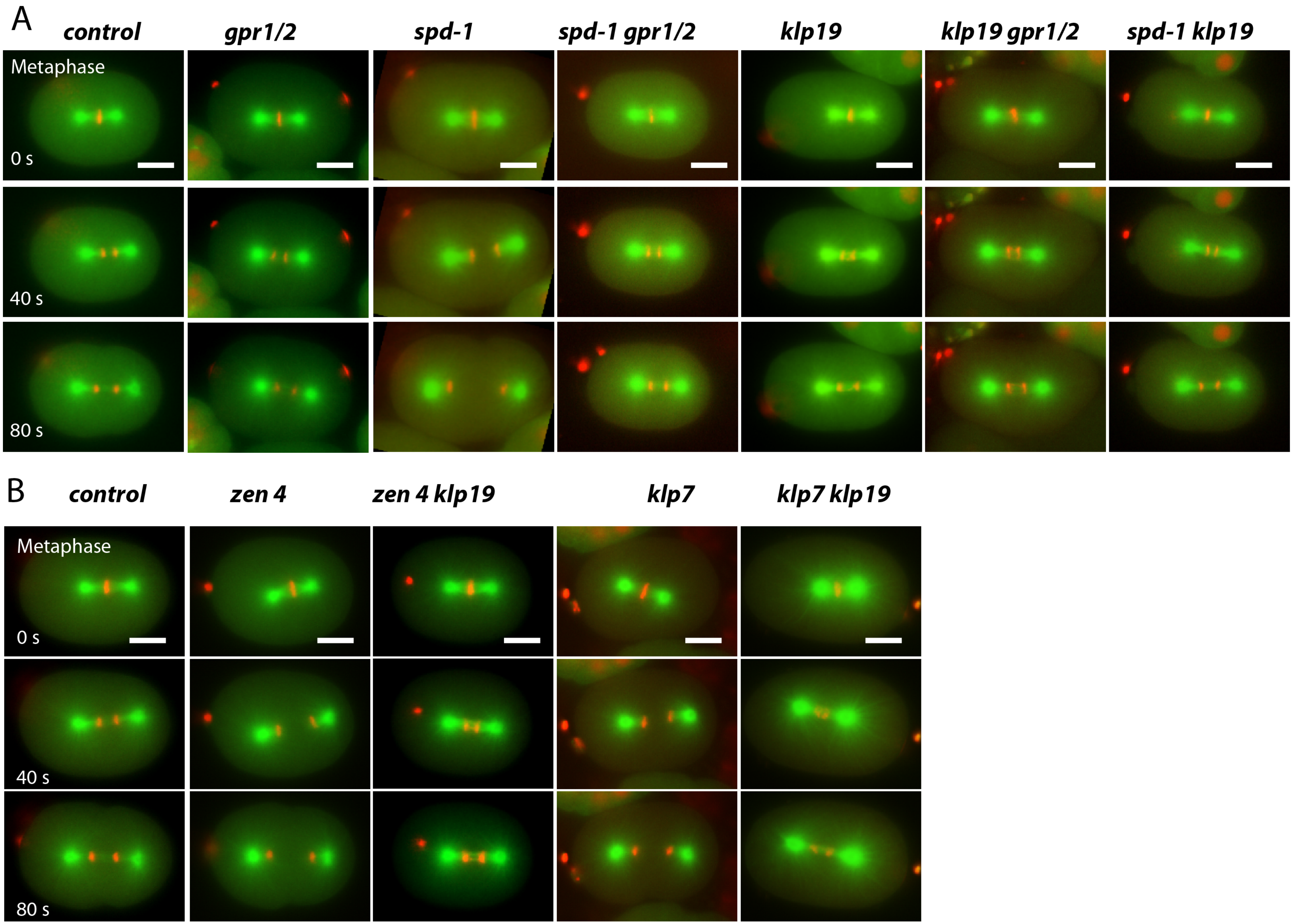

Supplementary figure 2

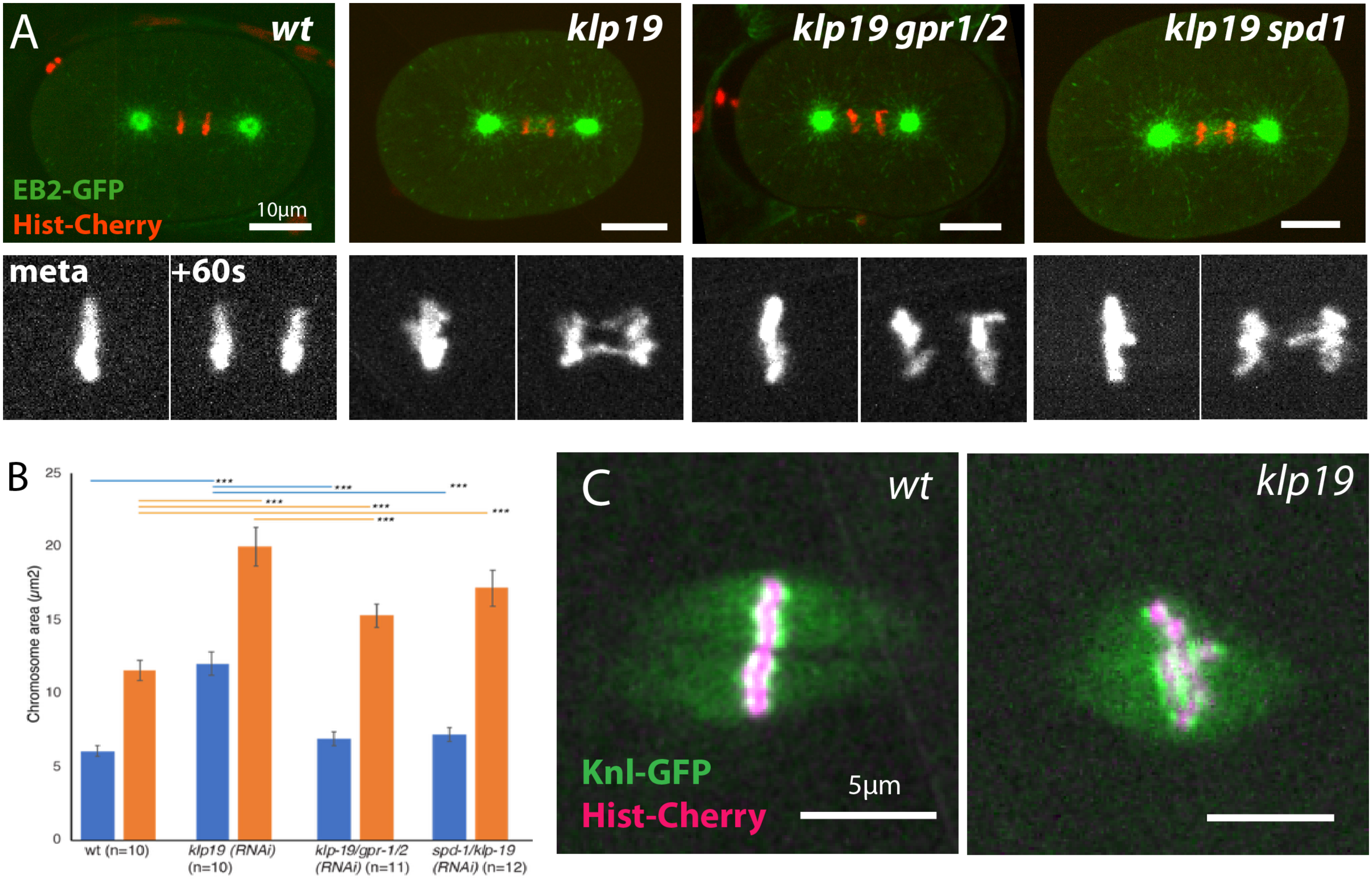

Supplementary figure 3

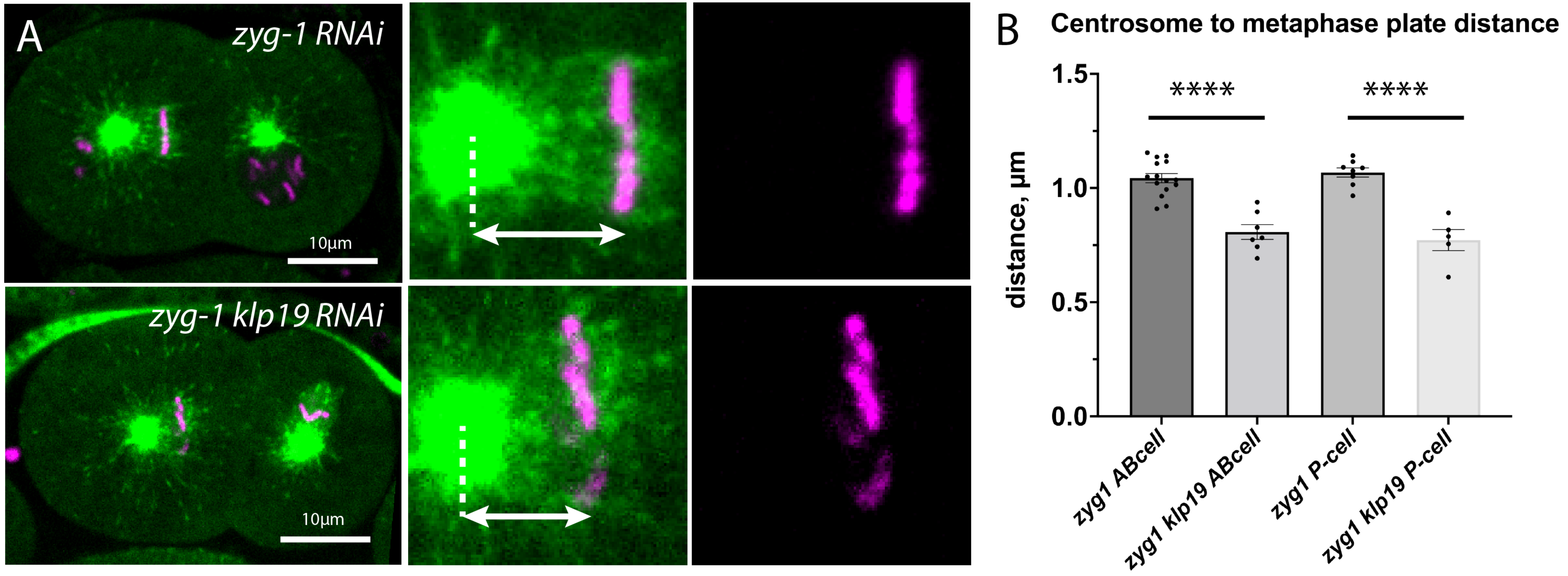

Supplementary figure 4

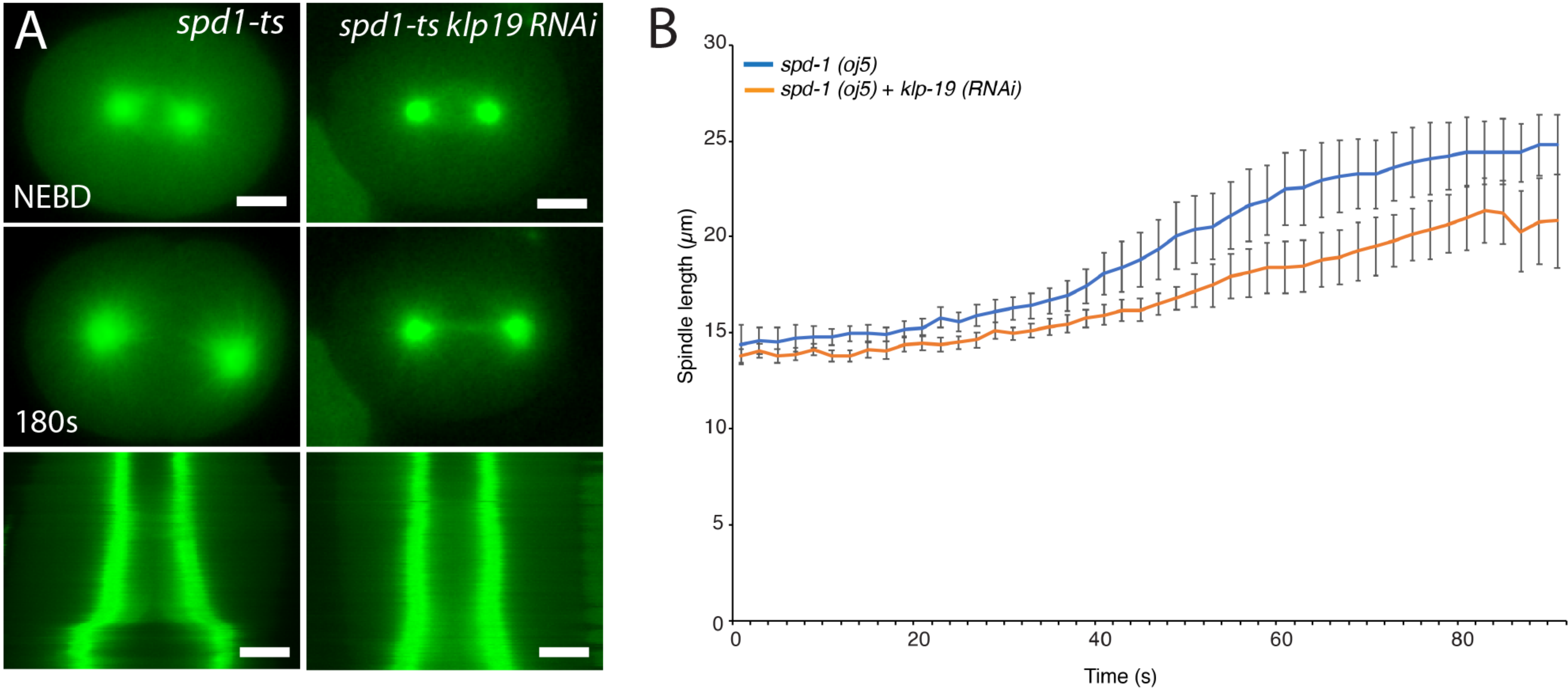

Supplementary figure 5

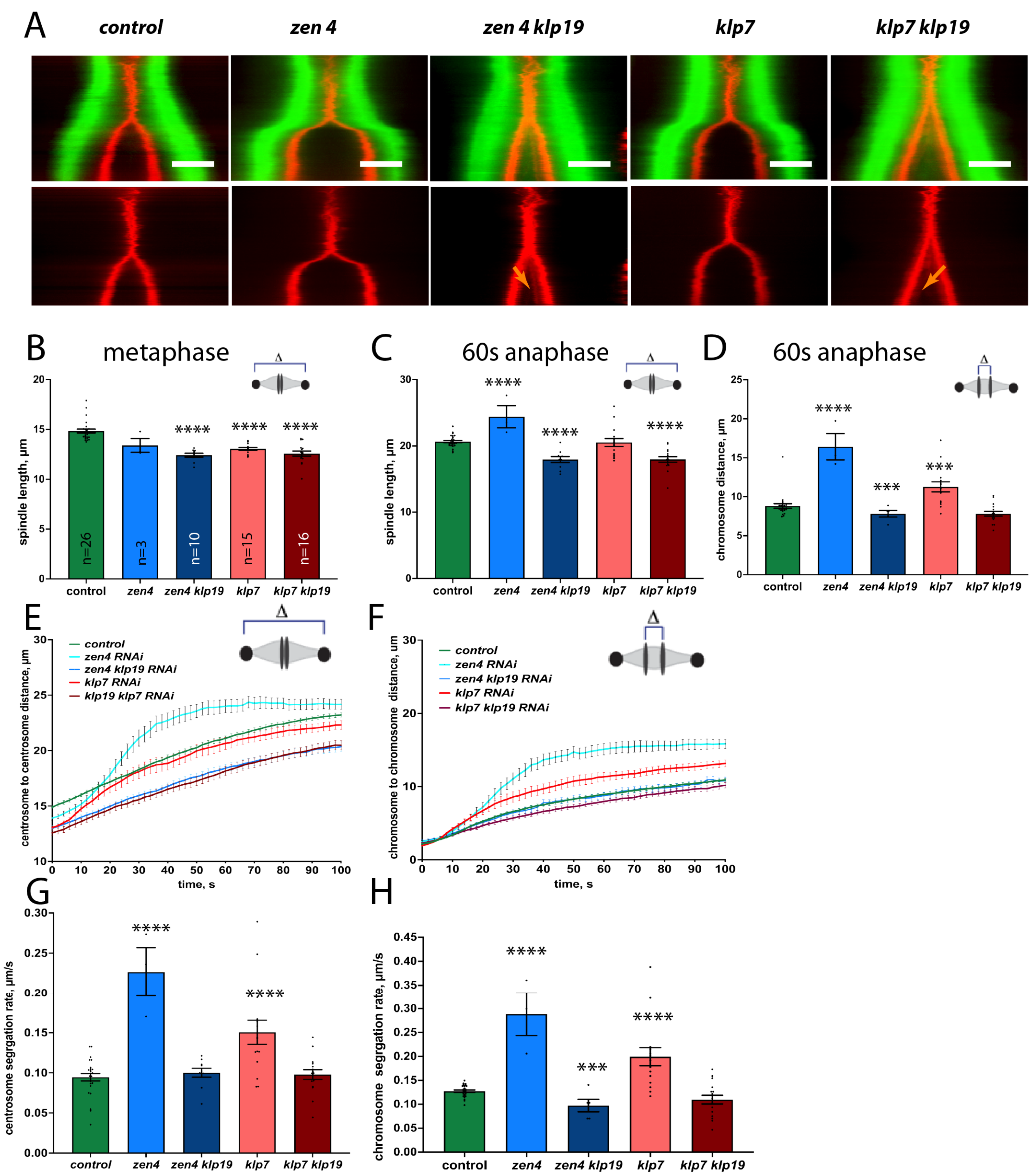

Supplementary figure 6

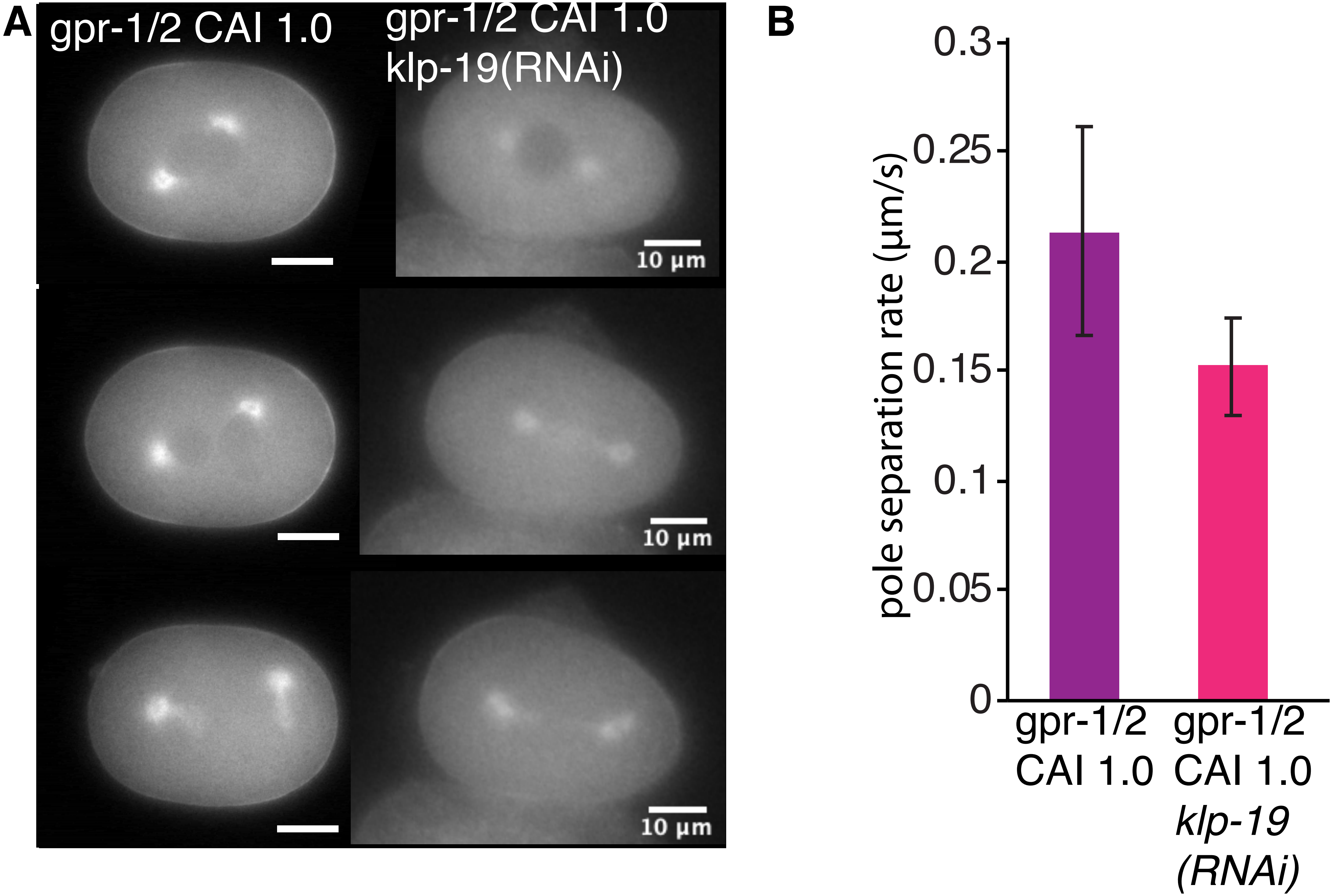

Supplementary figure 7

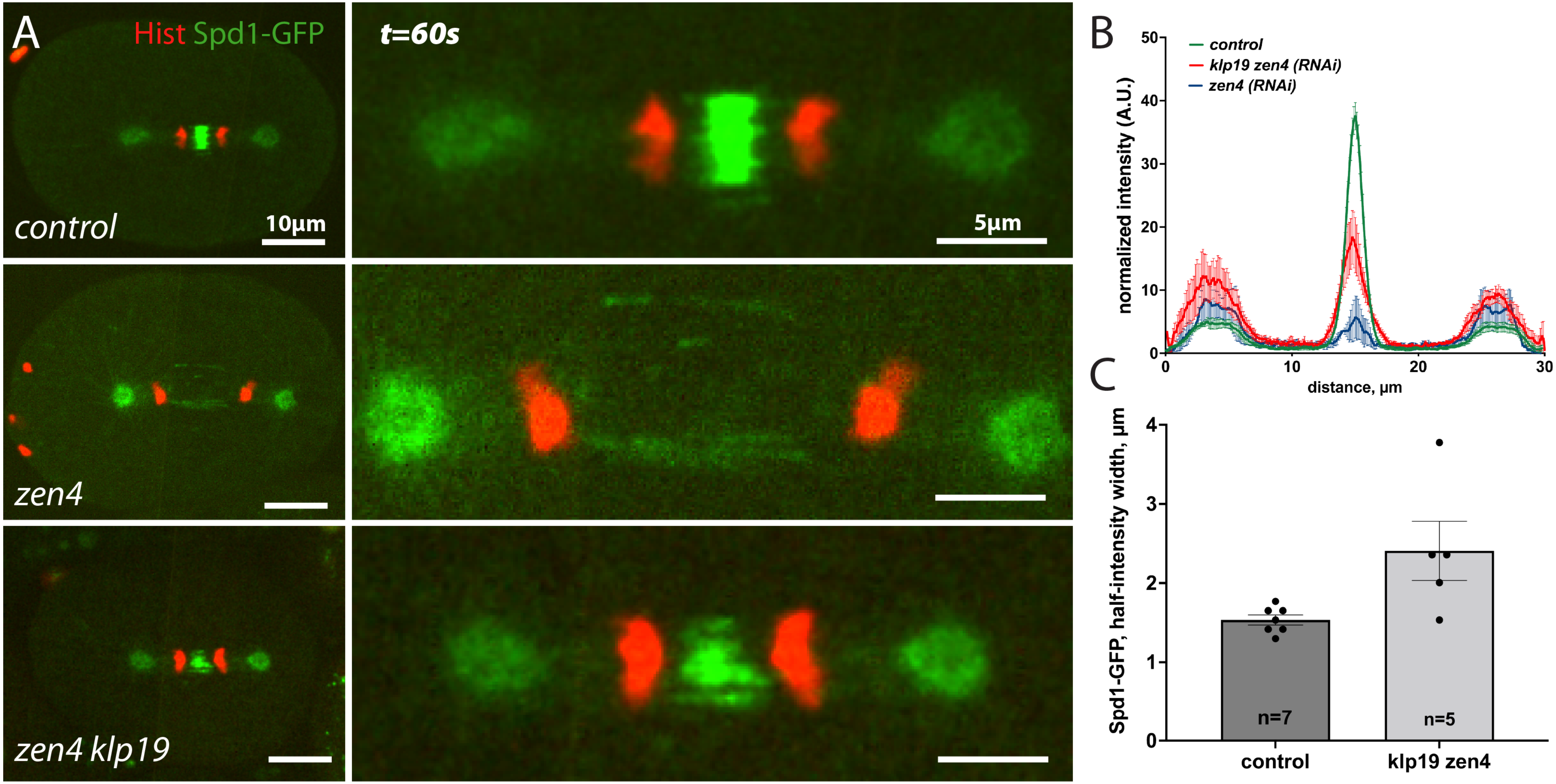

Supplementary Figure 8

A

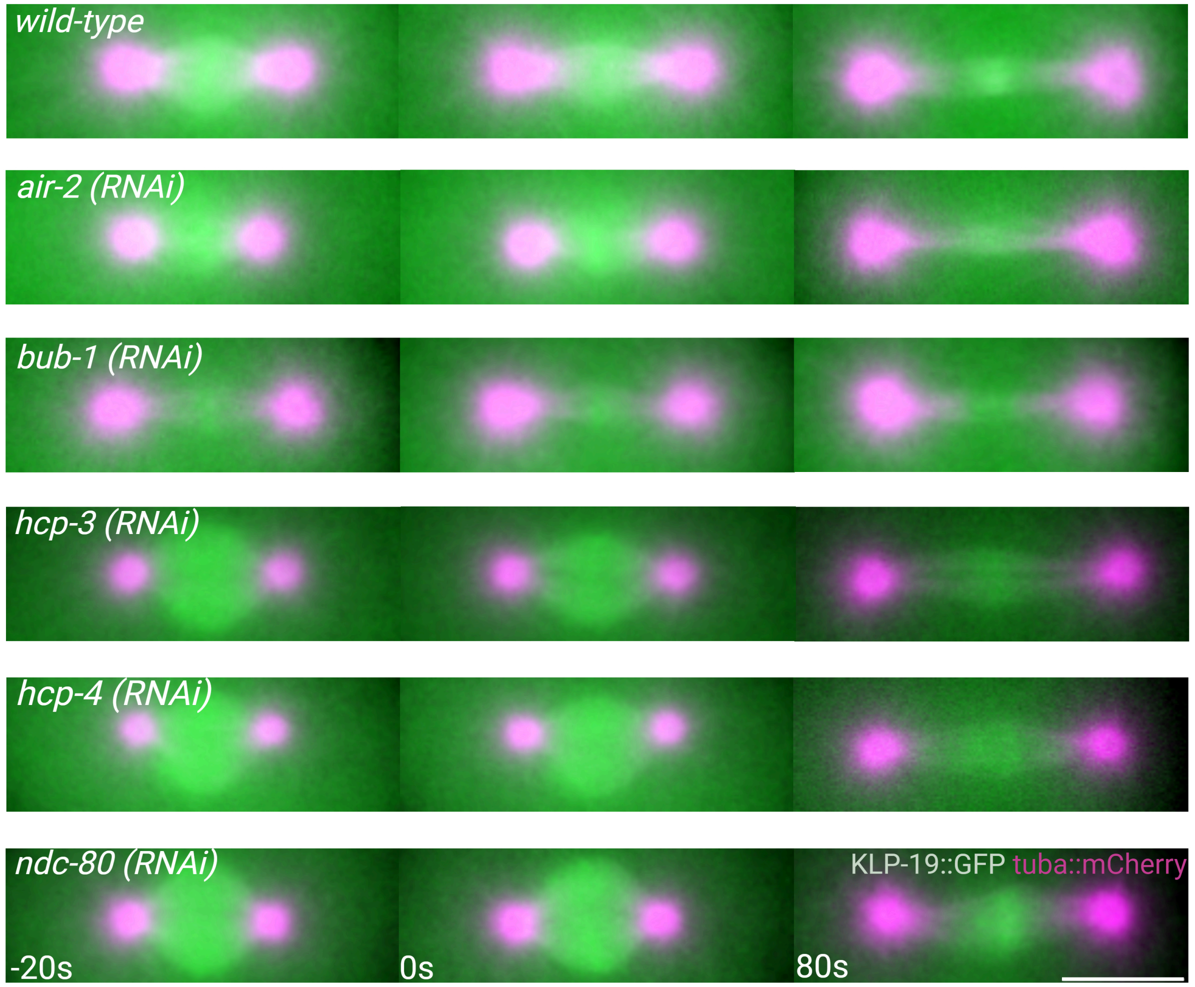

B

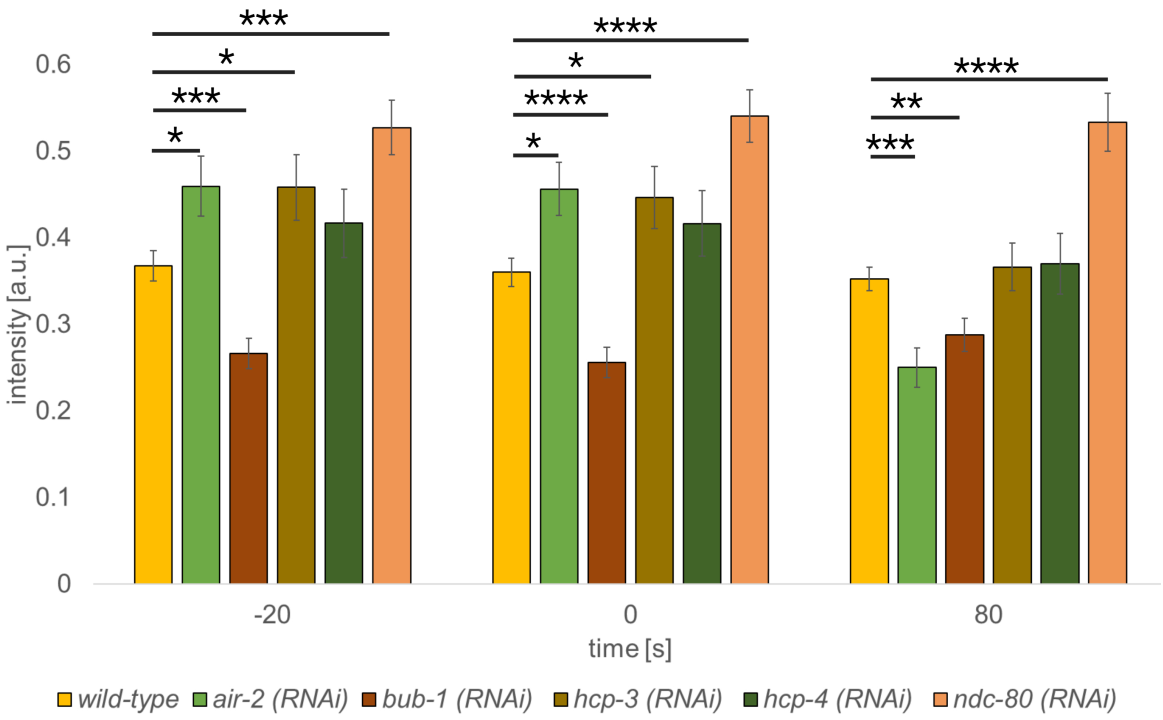

Supplementary Figure 9

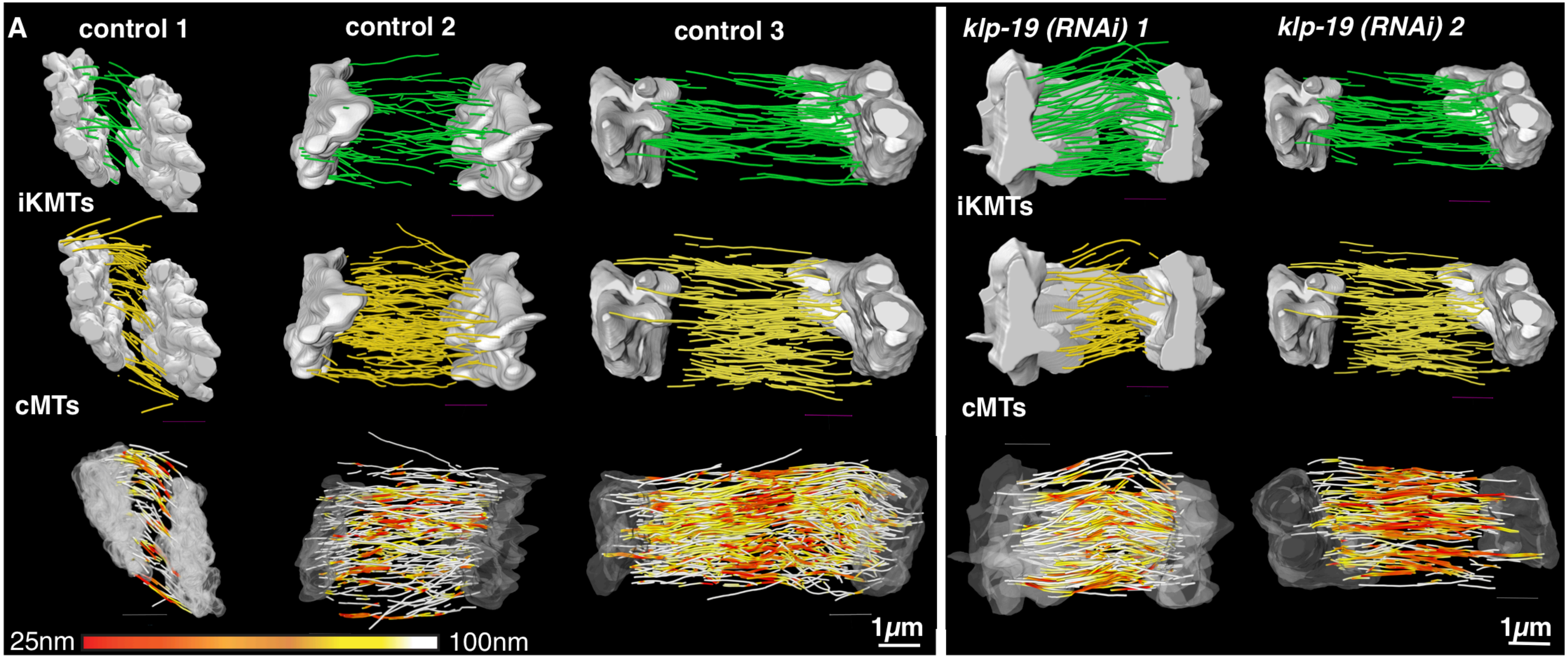

	Control 1	control 2	control 3	klp-19 (RNAi) 1	klp-19 (RNAi) 2
chrom dist μm	1.5	4	5.5	3	4.5
iKMT/μm^3	0.7	0.4	1.4	1.8	1.3
cMT/μm3	1.9	1.2	3.6	2.3	3
Av. MT Length μm	0.6 ± 0.03	0.95 ± 0.05	1.09 ± 0.04	1.12 ± 0.04	1.18 ± 0.05
AV. Interact. Length μm	0.39 ± 0.03	0.41 ± 0.01	0.46 ± 0.01	0.44 ± 0.01	0.56 ± 0.01
# Interact. per MT	0.4	1	3.3	3	1.6
Av. Interact. Distance nm	106 ± 6.6	83 ± 2.2	55 ± 1.3	62 ± 2.9	62 ± 2.3
